# Exploring electroencephalographic infraslow neurofeedback treatment for chronic low back pain: a double-blinded safety and feasibility randomized placebo-controlled trial

**DOI:** 10.1038/s41598-023-28344-2

**Published:** 2023-01-20

**Authors:** Divya Bharatkumar Adhia, Ramakrishnan Mani, Jerin Mathew, Finella O’Leary, Mark Smith, Sven Vanneste, Dirk De Ridder

**Affiliations:** 1 Department of Surgical Sciences, Otago Medical School, University of Otago, PO BOX 56, Dunedin, 9054 New Zealand; 2 Pain@Otago Research Theme, University of Otago, Dunedin, New Zealand; 3 Department of Physiotherapy, Manipal College of Health Professions, Manipal Academy of Higher Education, Manipal, India; 4 Centre for Health, Activity and Rehabilitation Research, School of Physiotherapy, University of Otago, Dunedin, New Zealand; 5 Neurofeedback Therapy Services of New York, New York, NY USA; 6 Global Brain Health Institute, Trinity College Dublin, Dublin, Ireland

**Keywords:** Neuroscience, Medical research, Pain

## Abstract

Chronic low back pain (CLBP) is a disabling condition worldwide. In CLBP, neuroimaging studies demonstrate abnormal activities in cortical areas responsible for pain modulation, emotional, and sensory components of pain experience [i.e., pregenual and dorsal anterior cingulate cortex (pgACC, dACC), and somatosensory cortex (SSC), respectively]. This pilot study, conducted in a university setting, evaluated the feasibility, safety, and acceptability of a novel electroencephalography-based infraslow-neurofeedback (EEG ISF-NF) technique for retraining activities in pgACC, dACC and SSC and explored its effects on pain and disability. Participants with CLBP (n = 60), recruited between July’20 to March’21, received 12 sessions of either: ISF-NF targeting pgACC, dACC + SSC, a ratio of pgACC*2/dACC + SSC, or Placebo-NF. Descriptive statistics demonstrated that ISF-NF training is feasible [recruitment rate (7 participants/month), dropouts (25%; 20–27%), and adherence (80%; 73–88%)], safe (no adverse events reported), and was moderate to highly acceptable [Mean ± SD: 7.8 ± 2.0 (pgACC), 7.5 ± 2.7 (dACC + SCC), 8.2 ± 1.9 (Ratio), and 7.7 ± 1.5 (Placebo)]. ISF-NF targeting pgACC demonstrated the most favourable clinical outcomes, with a higher proportion of participants exhibiting a clinically meaningful reduction in pain severity [53%; MD (95% CI): − 1.9 (− 2.7, − 1.0)], interference [80%; MD (95% CI): − 2.3 (− 3.5, − 1.2)], and disability [73%; MD (95% CI): − 4.5 (− 6.1, − 2.9)] at 1-month follow-up. ISF-NF training is a feasible, safe, and an acceptable treatment approach for CLBP.

## Introduction

Chronic low back pain (CLBP) is a significant and disabling health condition affecting individuals, the wider community, and the healthcare system^1^. General treatments for CLBP, include primarily pharmacological therapies; with increased risk of abuse, overdose, and other adverse outcomes (for example., with long term opioid therapy)^2–4^. Therefore, new non-pharmacological treatment approaches targeting mechanisms linked to CLBP need to be developed and evaluated for their clinical benefits.

Resting-state cortical activity alterations have been demonstrated in individuals with CLBP^5–8^. Evidence from a recent meta-analysis of functional imaging data suggests that chronic pain could result from an imbalance between cortical activity of regions that engages ascending nociceptive and descending (anti-nociceptive) inhibitory pathways^9,10^. The ascending nociceptive pathways consist of the lateral and medial pathways, involved in painfulness and suffering, respectively^9,10^. The most notably involved cortical areas that process painfulness and suffering includes primary somatosensory cortex (SSC), and dorsal anterior cingulate cortex (dACC), respectively^7,9–11^. The main hub of the descending pain inhibitory pathway includes pregenual anterior cingulate cortex (pgACC)^8–10^. It has been shown that if the balance between pain ascending and descending pathways as measured by the EEG source, localized current density equals 1, as computed by the current density of (dACC + SSC)/2*pgACC, that no pain is perceived, but that in neuropathic pain the balance > 1, i.e. there is more pain provoking activity in the dACC and SSC than pain inhibitory activity in the pgACC^10^. Furthermore, neuromodulatory interventions that target these pain processing brain regions (pgACC, dACC, and SSC) indirectly improve clinical outcomes. Indeed, previous studies using non-invasive transcranial direct current stimulation of the C2 dermatoma^12^ and invasive spinal cord stimulation^13^ have shown that these neuromodulatory approaches modulate the balance between the pain provoking and descending pain modulatory pathway. Yet the problem of these non-invasive and invasive neuromodulatory procedures is that the clinical benefit is dependent on the (intermittent) continuation of the neuromodulation. We therefore hypothesize that by using source localized neurofeedback, targeting these 3 areas directly, an improvement in pain perception can be achieved, and that by using an operant conditioning paradigm, typical of neurofeedback, this effect may last, as the brain is taught how to normalize its oscillations like pain-free levels.

Electroencephalography (EEG) based Neurofeedback (NF) is a brain-computer interface biofeedback technique that facilitates an individual’s ability to self-control their real-time cortical activity of the targeted brain regions and reinforces learning through operant conditioning^14^. Recent sLORETA based EEG-NF techniques, unlike traditional NF, implement the LORETA inverse solution algorithm in feedback calculation, thus increasing the spatial specificity and permitting training of multiple and specific brain regions simultaneously. In other words, a person can modify the electrical activity (e.g., power, coherence, asymmetries, phase-lag, and phase-reset) at the targeted brain regions in the desired direction through a closed-loop feedback system, in which an exogenous sensory stimulus (e.g., auditory tone) is fed back to the individuals in real-time following the attainment of the desired neural electrical activity, thus reinforcing learning. Previous EEG-NF studies, including case reports/non-randomised/open-label studies, targeted training higher frequency bands at specific electrode levels in individuals with chronic pain conditions^15–17^. Studies investigating the effect of EEG-NF training on CLBP outcomes are lacking; only two pilot studies have been reported to date^18,19^ targeting the alpha frequency at the sensor level rather than training specific brain regions at the source level. Higher frequency bands are believed to be nested on the infraslow frequency (ISF) bands (0.0–0.1 Hz)^20,21^. The ISF play a profound role in modulating and synchronizing high-frequency cortical activity^20–22^. Also, the ISFs are critically involved in mediating pain perception^23^. Recent evidence from imaging studies also demonstrates alterations in the ISF oscillations in individuals with CLBP in the pain processing brain regions (pgACC, dACC, SSC)^24,25^. Therefore, we hypothesise that the source localised EEG-NF specifically targeting the ISF bands in pain processing brain regions (pgACC, dACC, and SSC) could be more effective than targeting higher frequency bands at specific electrode levels and can potentially promote greater pain relief. To date, EEG ISF-NF training has been explored and demonstrated positive results by a few pilot studies as a potential treatment option for food addiction^26^, internalizing disorders^27,28^, and osteoarthritic pain^29^. However, none of the studies has explored EEG ISF-NF training as a potential treatment option for treatment of CLBP and is warranted given the high burden of CLBP. However, before conducting an adequately powered randomised controlled trial (RCT), it is essential to test the safety and feasibility of the proposed neurofeedback approach.

Therefore, this investigation explored the feasibility and safety of a novel source localised EEG-based ISF-NF training technique, targeting the pgACC and dACC + SSC regions for treating CLBP. The specific objectives of this study were to.

- to assess the feasibility, safety, and acceptability of the targeted EEG ISF-NF training in people with CLBP,
- to explore the immediate, intermediate, and short-term trends of the effects of the targeted ISF-NF training on pain and function.
- to explore the EEG changes [current density (CD) and functional connectivity (FC)] in the ISF band at the targeted brain regions following training.

We hypothesize that pgACC up-training will improve pain modulation through activation of descending pain inhibitory controls; while down-training medial (dACC) and lateral (SSC) pathways will disrupt pain-provoking activity; but that most benefit may be obtained by normalizing the pain provoking/pain inhibitory balance (up-training pgACC, and down-training dACC and SSC pathway simultaneously by ratio training).

A study is therefore set up to test these hypotheses and compare these three groups versus placebo-NF. The study results will support brain-computer interface training as a treatment tool for improving clinical outcomes in people with CLBP. This study will provide central tendency and variability data of clinical outcomes for estimating sample size for a full RCT.

## Methods

### Trial registration and ethical approval

Prospectively registered in Australian and New Zealand Clinical Trials Registry (https://www.anzctr.org.au/Trial/Registration/TrialReview.aspx?id=379470&isReview=true; Registration number: ACTRN12620000414910; Date of registration: 27/03/2020). The study was conducted according to the ethical standards of the 1964 Declaration of Helsinki. NZ Health and Disability Ethics Committee approved ethics (Ref:20/CEN/60).

### Study design

**Figure 1:**
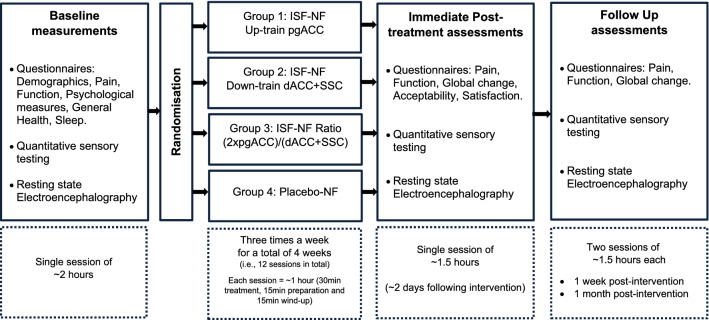
Study design and timelines. ISF-NF: infraslow frequency neurofeedback, pgACC: pregenual anterior cingulate cortex, dACC: dorsal anterior cingulate cortex, SSC: primary somatosensory cortex. Study assessment sessions were conducted at the Department of Surgical Sciences laboratory, Dunedin School of Medicine, Dunedin hospital, and the treatment sessions were conducted at the School of Physiotherapy laboratory, University of Otago, Dunedin, New Zealand. Feasibility measures were assessed throughout the study period. Treatment acceptability and satisfaction was assessed immediately post-intervention. Primary and secondary outcome measures and mechanistic outcome measure (EEG) were collected at baseline, immediately post-intervention, and at 1-week and 1-month post-intervention.

Double-blind, randomized, placebo-controlled feasibility study with four parallel intervention arms (Fig. 1). Feasibility and safety measures were collected throughout, while clinical measures and EEG were collected at baseline (T0), immediately (T1), 1 week (T2), and 1 month (T3) post-intervention.

#### Randomization

A research administrator, not involved in treatment/assessment, randomized, and assigned participants using a computerized open-access randomization software program without applying any restrictions (on a 1:1:1:1 basis) to either:

- Group-1: ISF-NF up-training pgACC, i.e., modulate the descending pain inhibitory pathway
- Group-2: ISF-NF down-training dACC + SSC, i.e., modulate the medial and lateral pathway
- Group-3: ISF-NF concurrently up-training pgACC and down-training dACC + SSC, i.e., Ratio [(2xpgACC)/(dACC + SSC)], i.e., normalize the balance between the descending inhibitory and ascending pain provoking pathways
- Group-4: Placebo-NF

The randomisation schedule was concealed in sequentially numbered, sealed opaque envelopes and provided to participants at baseline.

#### Blinding

Participants and outcome assessor were blinded. The success of blinding was assessed after completion of intervention using the question, “What type of treatment do you believe that you/the participant received respectively?” The confidence in their judgement was assessed on an 11-point NRS *(0* = *Not at all to 10* = *Extremely confident)*, with the reason being noted and whether the intervention was revealed to them.

### Participants and eligibility criteria

All participants were voluntarily recruited from the community through advertisement flyers. All participants provided written informed consent prior to study enrolment. Participants were screened for eligibility and enrolled by a musculoskeletal physiotherapy researcher.

#### Inclusion and exclusion criteria

Ages of 18 to 75 years, pain in the lower back region for ≥ 3 months, a score of ≥ 4 on an 11-point NPRS in 4 weeks prior to enrolment, a disability score of ≥ 5 on Roland–Morris Disability Questionnaire (RMDQ)^30^. Participants with the following were excluded: Inflammatory arthritis, auto-immune conditions, undergoing physiotherapists/chiropractic therapy, recent back injuries in ≤ 3 months, radicular pain/radiculopathy, spinal surgery/lumbar epidural injections in ≤ 6 months, current intake of centrally acting medications or intention of taking new medications in next 3 months, neurological diseases, substance abuse, dyslipidaemia, unstable medical/psychiatric conditions, epilepsy/seizures, peripheral neuropathy, vascular disorders, cognitive impairments, hearing problems, recent/current pregnancy, and presence of any electronic implants.

At baseline assessment, all participants completed questionnaires to capture demographics, and clinical characteristics of CLBP, including the presence of central sensitivity (Central Sensitization Inventory)^31^, neuropathic pain quality (PainDETECT)^32^, treatment expectancy/credibility^33^, sleep (Medical Outcomes Study-Sleep Scale)^34^, psychological measures (Depression, Anxiety, Stress Scale^35^, Pain Catastrophizing Scale^36^, Pain Vigilance Awareness Questionnaire^37^, Positive and Negative Affect Schedule-short form^38^, Emotion Regulation Questionnaire^39^, Five-Facet Mindfulness Questionnaire-15^40^) and general well-being (European Quality of Life^41^ and WHO-Five Well-Being Index^42^).

#### Sample size

As this was a pilot study to determine the feasibility of a future fully powered RCT, sample size calculation was not performed. Based on statistical advice, a sample of 60 participants (15/group) was considered enough to determine feasibility issues and obtain treatment estimates for designing the full trial.

### Intervention

Source localised EEG ISF-NF was administered three times a week (30 min/session) for four consecutive weeks (12 sessions) by the researcher (physiotherapy background) experienced in delivering neuromodulation techniques. Treatment was delivered using a 21-channel DC coupled amplifier and BrainAvatar™ sLORETA software version 4.7.5 for Discovery manufactured by BrainMaster Technologies Inc., Bedford, OH, USA^43^. The sLORETA source localization permits the selection of any region of the brain for feedback of CD, using voxels as regions of interest (ROI), which are selected based on MNI coordinates. The CDs for chosen voxels are computed continuously using Fast Fourier Transformation and inverse solution sLORETA software for targeted brain regions and can be fed back to participants by using sound feedback.

**Figure 2:**
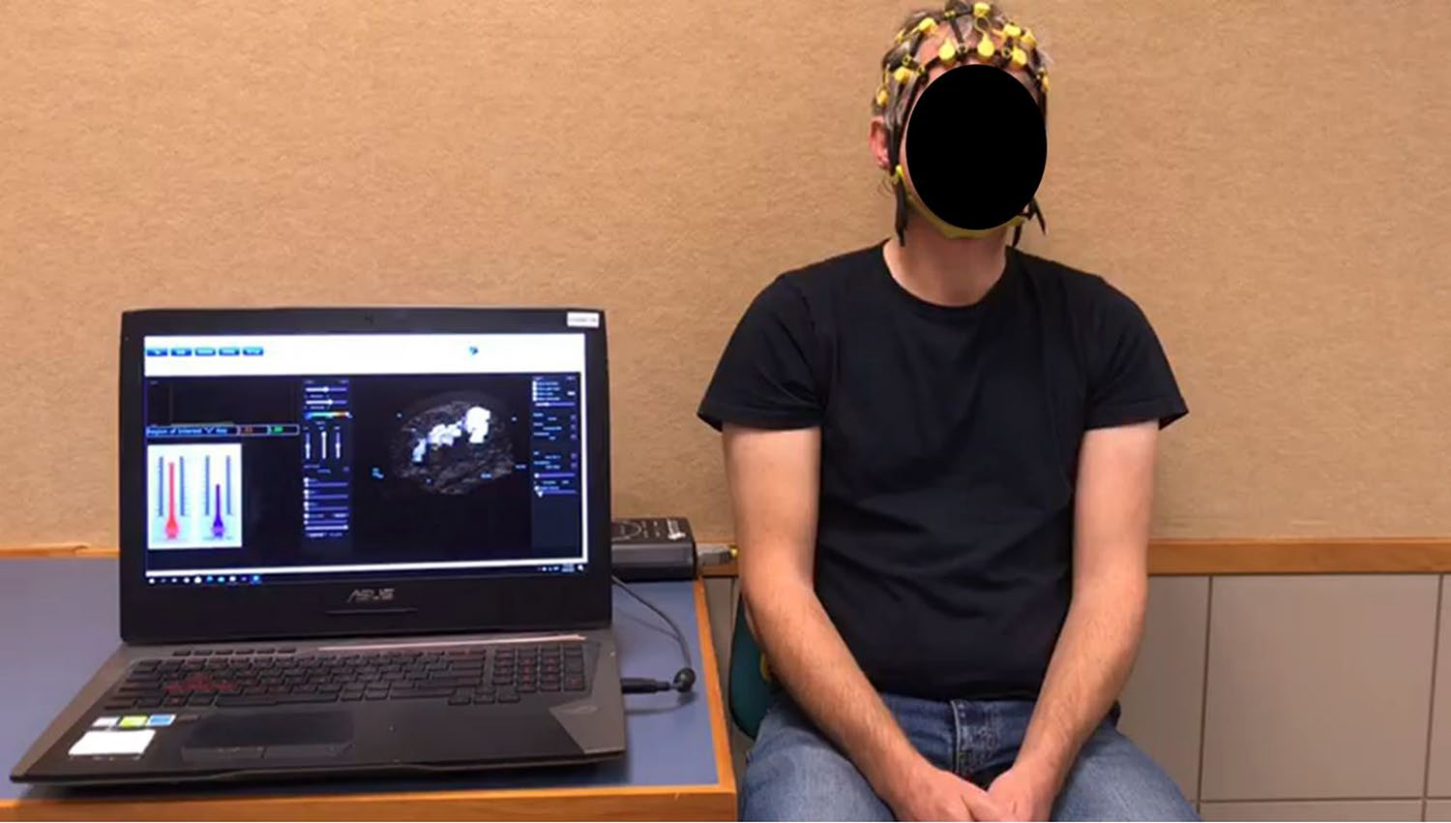
EEG ISF-NF intervention set-up.

During each session, the Comby EEG lead cap with 19 (Ag/AgCl) electrodes positioned according to the International 10–20 system was fixed to individual’s scalp (Fig. 2). The impedance of electrodes was monitored and kept below 5 kΩ. Participants were instructed to close their eyes, relax, minimize movements, and listen to sound feedback. The system delivers sound feedback (reward) each time participant's brain activity meets the desired infraslow (0.0–0.1 Hz) threshold at targeted brain regions. No explicit instructions regarding mental strategies to be used during NF training were provided. Mood, engagement, and motivation levels were assessed at every training session, using NRS.

#### ISF-NF treatment groups

It has been demonstrated that chronic pain can be considered as an imbalance between pain input and pain suppression. Our protocols are derived from this theory utilizing source localization neurofeedback targeting the ratio between pgACC, SSC, and dACC. For the current study, we developed EEG-NF training programs to up-train (i.e., increase CD) ISF activity at pgACC (Group 1), and down-train (i.e., decrease CD) ISF activity simultaneously at dACC and SSC (Group 2). For Group 3, a program was developed to concurrently up-train ISF activity at pgACC (× 2) and down-train ISF activity at dACC + SSC, to reinforce ratio between these regions to be > 1, as below:

$$\mathrm{Ratio}=\frac{2\mathrm{xpgACC}}{\mathrm{dACC}+\mathrm{SSC}}>1$$

For all groups, the reward threshold was adjusted in real-time between 60 and 80%, i.e., for 60–80% of time, sound feedback was delivered by system when participant's brain activity meets desired infraslow magnitude.

#### Placebo-NF group

To create identical auditory feedback to ISF-NF groups, participants in placebo-NF group listened to a random set of pre-recorded sound files (n = 12), sourced from a database of recorded audio files (using audacity software) of healthy participants that underwent EEG source localised ISF-NF training (targeting ratio between pgACC and dACC + SSC). All other conditions were kept same as ISF-NF groups.

### Outcome measures

#### Feasibility measures

Included recruitment rate (number of participants recruited per month), proportion of participants recruited from total number screened (expressed as a percentage), adherence to intervention (measured as number of treatment sessions attended by each participant expressed as a percentage of the total number of sessions), and dropout rates (measured as the number of participants who dropped out in each group, expressed as a percentage of the total number of participants enrolled in the study).

#### Safety measures

Adverse effects and related dropouts were recorded. The Discontinuation-Emergent Sign and Symptom (DESS) scale were used to record deterioration in any side effects compared to the status prior to the previous session and record any new symptoms^44^. An independent Data and Safety Monitoring Committee monitored the safety of the study. A serious adverse event (SAE) was defined as any untoward medical occurrence or effect that results in death, is life-threatening, requires hospitalisation, and results in persistent or significant disability or incapacity. It was planned that the study would be discontinued if there was any unexpected SAE, or other unexpected events, if funding was completed/insufficient, or if the desired sample size was reached.

*Intervention acceptability and satisfaction* were recorded on NRS (0 = Not at all acceptable/satisfied to 10 = Very acceptable/satisfied). Qualitative written feedback about participant experiences of NF treatment was also obtained using open-ended questions.

#### Clinical measures

Brief Pain Inventory (BPI)^45^, a valid and reliable tool [test–retest reliability (0.97), internal consistency for pain severity (0.82) and interference (0.93), validity: high correlations with RMDQ (0.57–0.81), responsiveness: SRM for pain severity (− 1.09) and interference (− 1.13)]^46^, was used to capture pain intensity and interference in daily activities. Pain unpleasantness and bothersomeness were measured using NRS (0 = not unpleasant/bothering to 10 = most unpleasant/bothering imaginable)^47^ [test–retest reliability (0.97), validity: high correlations with VAS (0.81), responsiveness: SRM (1.1)]^48^. RMDQ^30^ [test–retest reliability (0.86), internal consistency (0.86), validity: fair correlations with Quebec Back Pain Disability Scale (0.6), responsiveness: ROC (0.77)]^46^ was used to assess self-reported functional abilities. The self-perceived global rate of change^49^ was assessed using question, *“Compared to the beginning of treatment, how would you describe your back at this moment?”*; rated on an 11-point scale (− 5 = much worse, through 0 = unchanged, to + 5 = completely recovered).

#### Measures of peripheral and central sensitization

Quantitative sensory testing (QST) was conducted according to guidelines^50,51^ and our previous study^52^, for symptomatic low back and non-dominant wrist regions in random order.

- *Mechanical temporal summation (MTS):* was assessed using a nylon monofilament (Semmes monofilament 6.65, 300 g). Brief ten repetitive contacts were delivered at 1 Hz rate, externally cued by auditory stimuli. Participants rated pain intensity on NRS immediately after the first contact, followed by rating their greatest pain intensity after 10th contact. MTS was calculated as difference between two NRS ratings (post–pre). Average of three trials were used for analysis^52^.
- *Pressure pain threshold (PPT):* A computerized, handheld digital algometer (AlgoMed-Medoc) was used. Two familiarization trials were performed at dominant mid-forearm. Algometer probe (1 cm^2^) was pressed over marked test site perpendicularly to skin at 30 kPa/s. Participants pressed trigger button when pressure sensation changed to first pain, and the amount of force was recorded. Average of three trials was used for analysis.
- *Condition pain modulation (CPM)* was administered 20 min after MTS and PPT procedures^53^.
  Conditioning stimulus consisted of cold pressor task. Participants immersed their dominant hand in a thermos containing circulating cold water (~ 5 °C) for 2 min or until it was too uncomfortable (NRS ~ 80%).
  Test stimulus: Algometer was used to measure suprathreshold PPT (pain40) at non-dominant leg region (tibialis anterior muscle). Two trials were recorded before conditioning stimulus and averaged to obtain a baseline score. Three PPT (pain40) trials were recorded in same region at 30, 60, and 90 s immediately after conditioning stimulus.
  A percent change score was calculated for each time point (CPM30sec, CPM60sec, and CPM90sec), with a positive score indicating an increase in PPTs after conditioning stimulus and thus presence of CPM effect.
  $${\text{CPM}}\;{\text{percent}}\;{\text{change}}\;{\text{score}} = \frac{{{\text{Postscore}} - {\text{Prescore}}}}{{{\text{Prescore}}}} \times 100$$

### Electroencephalogram

Resting-state eyes-closed EEG (~ 10 min) was obtained using SynAmps-RT Amplifier (Compumedics-Neuroscan). Sixty-four electrodes were placed in 10–10 International placement and impedances were checked to remain below 5kΩ. Data were resampled (128 Hz), band-pass filtered (0.005–0.2 Hz), plotted in EEGLAB and ICoN software for careful inspection and manual artefact rejection. SLORETA source localisation software was used to estimate intracerebral electrical sources that generates scalp-recorded activity. We calculated average fourier cross-spectral matrices for three ISF bands: ISF1 (low:0.01–0.04 Hz), ISF2 (mid:0.05–0.07) and ISF3 (high:0.08–0.10). Log-transformed CDs and lagged phase coherence (FC) were calculated for and between targeted ROIs (pgACC, dACC, and left and right SSC) respectively.

### Data analysis

Data were analysed using SPSS_v27.0. As this was a feasibility study, tests for significance to compare clinical and EEG measures between study groups were not performed, but descriptive statistics were applied. Feasibility outcomes are reported based on recommendations.

Clinical outcomes were analyzed based on intention-to-treat principle and as per the originally assigned groups. Last observation carried forward methodology was used to impute missing data. Mean ± SDs and Mean differences (95% CI), were calculated from baseline to each interim and primary endpoint (T3) for all clinical and experimental pain measures, and descriptively compared between groups.

Percentage change to baseline was calculated for primary pain (BPI) and functional (RMDQ) measures as below (e.g., for T3):

$${\text{Percent}}\;{\text{change}}\;{\text{to}}\;{\text{baseline}} = \frac{{{\text{T}}3 - {\text{T}}0}}{{{\text{T}}0}} \times 100$$

A ≥ 30% decrease was considered as meaningful clinical important difference (MCID). Proportion of participants with changes ≥ MCID were calculated and descriptively compared between groups.

Similarly, EEG measures (CD and FC) were also analysed descriptively and compared between groups.

### Protocol changes

Following changes were made to the registered protocol based on the ethical review and the peer reviewer comments. *Eligibility criteria:* The age bracket for participant inclusion was expanded to 18 to 75 years instead of the originally planned 35 to 70 years. *Secondary outcomes:* The MTS and PPT tests were evaluated at two sites (symptomatic low back and non-dominant wrist region) rather than the originally planned three regions (i.e., symptomatic low back region, non‐symptomatic low back region, and the distant non‐dominant wrist). Also, for the CPM procedure, the test site was changed to the non-dominant leg region, rather than the originally planned most painful low back region. All these changes to the protocol were made before the participant enrolment commenced., and are updated in the ANZCTR trial registry (https://www.anzctr.org.au/Trial/Registration/TrialReview.aspx?id=379470&isReview=true). The full trial protocol will be available from the corresponding author on reasonable request.

## Results

### Participants

**Figure 3:**
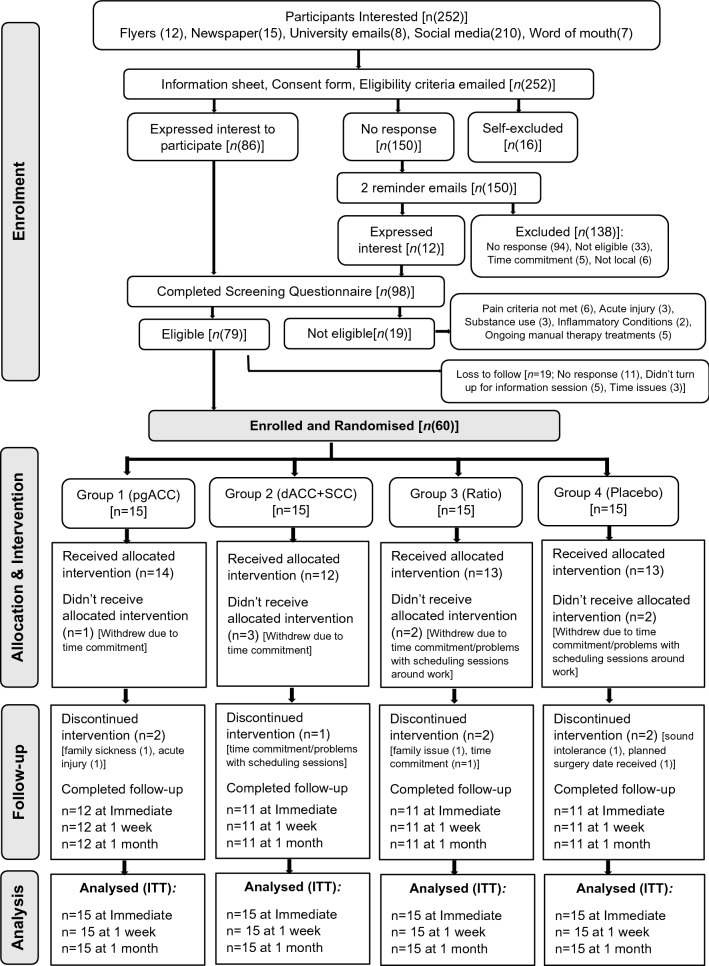
Flow of participants through the study phases. pgACC: pregenual anterior cingulate cortex, dACC: dorsal anterior cingulate cortex, SSC: primary somatosensory cortex, ITT: intention to treat.

Sixty participants were enrolled and randomised equally into four treatment groups (Fig. 3). Table 1 presents descriptive data for all participants at baseline, indicating all groups were comparable.

**Table 1:** Demographics and clinical characteristics of participants.

Characteristics/measures	Group 1 (pgACC) (n = 15)	Group 2 (dACC + SSC) (n = 15)	Group 3 (Ratio) (n = 15)	Group 4 (Placebo) (n = 15)
Age (years)	41.9 ± 15.8	39.9 ± 15.4	43.9 ± 15.4	42.5 ± 15.4
Sex				
Female; n (%)	9 (60)	13 (87)	10 (67)	11 (73)
Male; n (%)	6 (40)	2 (13)	5 (33)	4 (27)
Ethnicity				
NZ European; n (%)	11 (73)	7 (47)	11 (73)	9 (60)
Maori; n (%)	0 (0)	2 (13)	0 (0)	3 (20)
Indian; n (%)	0 (0)	1 (7)	0 (0)	0 (0)
Chinese; n (%)	1 (7)	2 (13)	0 (0)	1 (7)
Other; n (%)	3 (20)	3 (20)	4 (27)	2 (13)
Employment				
Employed; n (%)	8 (53)	6 (40)	8 (53)	7 (47)
Unemployed; n (%)	0 (0)	1 (7)	4 (27)	2 (13)
Retired; n (%)	1 (7)	2 (13)	1 (7)	1 (7)
Looking after family; n (%)	0 (0)	0 (0)	0 (0)	0 (0)
Self-employed; n (%)	2 (13)	2 (13)	0 (0)	4 (27)
Other; n (%)	4 (27)	4 (27)	2 (13)	1 (7)
Education				
University degree; n (%)	5 (33)	10 (67)	10 (67)	7 (47)
Trade/Apprenticeship; n (%)	3 (20)	1 (7)	1 (7)	2 (13)
Certificate/Diploma; n (%)	4 (27)	2 (13)	1 (7)	2 (13)
Year 12/quivalent; n (%)	3 (20)	1 (7)	1 (7)	3 (20)
Year 10/equivalent; n (%)	0 (0)	1 (7)	2 (13)	0 (0)
No formal qualification; n (%)	0 (0)	0 (0)	0 (0)	1 (7)
Neuropathic pain (PainDetect) (Mean ± SD)	10.9 ± 7.2	11.7 ± 6.2	11.6 ± 7.7	8.8 ± 4.1
Central sensitisation (CSI) (Mean ± SD)	41.3 ± 19.6	39.3 ± 17.2	41.5 ± 10.0	37.4 ± 14.2
Well-being (WHO-5)	13.5 ± 4.8	14.1 ± 3.7	12.9 ± 4.6	15.2 ± 4.6
Quality of life (EQ-5D)				
Index score (Mean ± SD)	0.6 ± 0.3	0.7 ± 0.1	0.7 ± 0.2	0.8 ± 0.1
VAS (Mean ± SD)	75.3 ± 18.6	69.9 ± 17.0	63.3 ± 20.1	76.0 ± 17.3
Sleep (MOS-Sleep)				
Index I (Mean ± SD)	45.3 ± 26.4	36.7 ± 18.3	40.7 ± 15.0	33.3 ± 15.0
Index II (Mean ± SD)	49.3 ± 26.6	40.4 ± 18.0	42.6 ± 15.7	37.3 ± 18.1
Pain catastrophising (PCS)				
Rumination (Mean ± SD)	5.5 ± 4.1	6.1 ± 3.3	4.4 ± 2.3	5.6 ± 4.0
Magnification (Mean ± SD)	3.4 ± 2.6	2.1 ± 1.7	3.1 ± 2.1	3.5 ± 2.9
Helplessness (Mean ± SD)	7.0 ± 4.1	5.5 ± 3.4	6.6 ± 4.3	7.1 ± 6.1
Total (Mean ± SD)	15.9 ± 9.8	13.7 ± 7.2	14.1 ± 7.5	16.1 ± 12.2
Pain vigilance and awareness (Mean ± SD)	40.6 ± 13.2	37.5 ± 14.9	41.4 ± 9.7	41.9 ± 11.0
Depression (DASS-21) (Mean ± SD)	4.3 ± 5.1	4.3 ± 3.8	3.9 ± 3.7	3.0 ± 2.5
Anxiety (DASS-21) (Mean ± SD)	3.9 ± 3.7	3.7 ± 3.5	3.4 ± 2.8	3.7 ± 2.9
Stress (DASS-21) (Mean ± SD)	7.1 ± 5.0	5.9 ± 3.6	7.7 ± 2.6	7.1 ± 3.8
Positive Affect (PANAS) (Mean ± SD)	30.7 ± 5.3	31.9 ± 6.1	28.1 ± 5.7	30.0 ± 5.2
Negative Affect (PANAS) (Mean ± SD)	18.6 ± 7.6	17.4 ± 6.2	19.3 ± 5.8	18.6 ± 5.1
Emotional regulation (ERQ)				
Cognitive reappraisal (Mean ± SD)	27.0 ± 8.4	31.7 ± 5.4	30.0 ± 6.3	28.1 ± 5.7
Emotional suppression (Mean ± SD)	14.4 ± 5.7	12.5 ± 3.9	15.2 ± 4.9	14.9 ± 4.5
Treatment expectation				
Credibility (Mean ± SD)	23.6 ± 20.2	17.9 ± 5.2	19.7 ± 5.7	18.7 ± 5.6
Expectancy (%) (Mean ± SD)	50 ± 40	49 ± 29	47 ± 22	44 ± 24
Mindfulness (FFMQ) (Mean ± SD)	47.7 ± 8.1	51.5 ± 7.6	51.1 ± 7.2	49.2 ± 6.2

### Feasibility

#### Recruitment

The total recruitment period was 9 months (July 2020 to March 2021), with the last follow up completed in May 2021. This feasibility trial was stopped in May 2021 as the desired sample size was reached (n = 60) and all follow ups completed. Our average recruitment rate was seven participants per month. The proportion of participants recruited (n = 60) from the total number of participants screened (n = 252) was 24%, which was greater than our a priori criteria of 20% (Fig. 3).

#### Dropouts

Of the total participants enrolled (n = 60), we lost 8 participants following baseline assessment session (Fig. 3). The most common reason for dropouts was time commitment required and fitting treatment sessions around participant’s work schedule. Further, seven participants discontinued treatment due to various reasons (outlined in Fig. 3). Thus, the overall dropout rate was 25% (n = 15 participants), which was less than our a priori criteria of 30%. The dropout rate for Group 1 was 20% and for Groups 2, 3, and 4 was 27% respectively (Fig. 3).

#### Treatment adherence and engagement

The average treatment adherence rate for all groups was 80%. The individual treatment adherence scores were 88%, 73%, 76%, and 81% for pgACC, dACC + SSC, Ratio, and Placebo groups respectively.

The NRS scores for mood, motivation, and treatment engagement were comparable between treatment groups. The Median (95% CI) for the pgACC, dACC + SSC, Ratio, and Placebo group for mood were 7.2 (6.0,7.8), 7.7 (5.3,8.3), 7.0 (6.0, 7.9), and 7.0 (6.9, 9.0) respectively; for motivation were 6.7 (5.6,7.5), 7.1 (5.2,7.8), 7.0 (5.3,8.3), and 7.0 (6.5,9.0) respectively; and for treatment engagement were 7.8 (5.7,8.3), 7.3 (5.0,8.3), 7.7 (6.8,8.8), and 7.7 (7.0,7.9) respectively. Overall, irrespective of the treatment group, participants reported moderate to high levels of mood, motivation, and engagement during NF training sessions.

#### Integrity of blinding

Participant blinding was deemed successful as the treatment group was not revealed to them in any way. In total, 51% of participants incorrectly predicted the treatment group or responded, “don’t know”. The remaining 49% of participants, although correctly predicted their treatment groups, based their decision primarily on guesswork or symptom assessment, and their confidence for correctly predicting the group was not greater than merely chance [Mean ± SD (48% ± 19%)]. Outcome assessor blinding was highly successful, with correct prediction being only 18%, and 58% of responses being “don’t know”.

### Adverse effects

**Figure 4:**
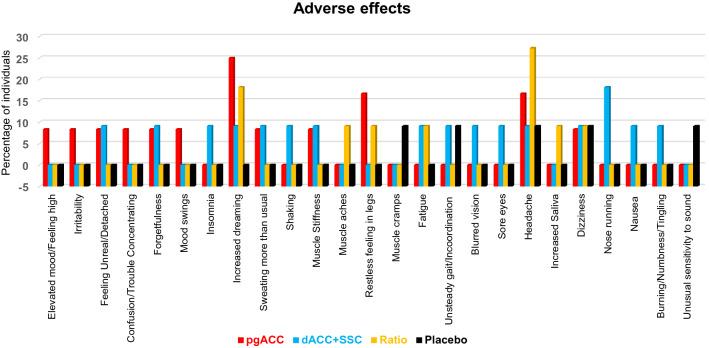
Adverse effects reported by participants during the neurofeedback treatment sessions. pgACC: pregenual anterior cingulate cortex, dACC: dorsal anterior cingulate cortex, SSC: primary somatosensory cortex.

No serious adverse effects were reported. Several transient low intensity (< 3 on NRS) negative side effects, rated to be related to ISF-NF treatment, were reported by a few participants (Fig. 4). The most common side effects in treatment groups included mild headaches and increased dreaming. Only one participant in Placebo-NF group discontinued the intervention due to intolerance to sound feedback (Fig. 3).

### Acceptability and satisfaction

All participants, irrespective of treatment group, reported moderate to high levels of acceptability with Mean ± SD of 7.8 ± 2.0 (pgACC), 7.5 ± 2.7 (dACC + SCC), 8.2 ± 1.9 (Ratio), and 7.7 ± 1.5 (Placebo), respectively. Further, moderate to high levels of satisfaction were also reported with Mean ± SD of 5.7 ± 2.9 (pgACC), 7.3 ± 2.5 (dACC + SCC), 7.5 ± 2.4 (Ratio), and 7.0 ± 1.5 (Placebo), respectively. Summary of qualitative feedback to open-ended questions is presented in Supplementary Table S1 online.

### Clinical measures

**Figure 5:**
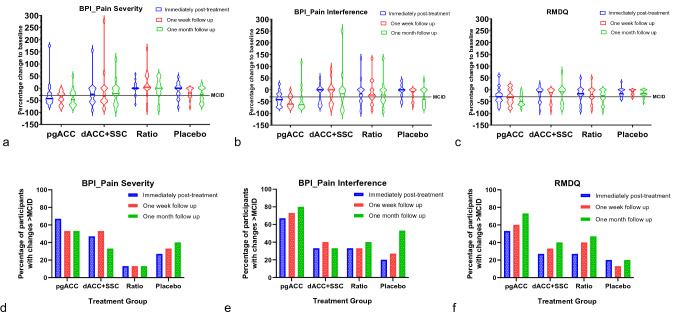
Violin plots for percentage change to baseline in pain severity (**a**), pain interference (**b**), and disability (**c**), and the proportion of participants demonstrating clinically meaningful reductions in pain severity (**d**), pain interference (**e**), and disability (**f**) expressed as percentage. pgACC: pregenual anterior cingulate cortex, dACC: dorsal anterior cingulate cortex, SSC: primary somatosensory cortex, BPI: Brief pain inventory, RMDQ: Roland Morris Disability Questionnaire, MCID: Minimal clinically important difference. The thick line in each of the violine plots (**a**–**c**) represents the median.

Tables 2 and 3 presents descriptive data for primary and secondary pain and functional measures respectively. Figure 5 presents the violin plots for percentage change to baseline for primary clinical measures of pain (BPI severity and interference sub score) and function (RMDQ).

**Table 2:** Descriptive data for the primary pain and functional measures at all timepoints.

Variable	Time point	Group 1 (pgACC) (n = 15)	Group 2 (dACC + SSC) (n = 15)	Group 3 (Ratio) (n = 15)	Group 4 (Placebo) (n = 15)
BPI: Pain severity Mean ± SD MD (95% CI)	T0	4.2 ± 1.8	3.3 ± 1.6	3.4 ± 1.3	3.6 ± 1.4
	T1	2.7 ± 1.7	2.4 ± 1.5	3.5 ± 1.4	3.3 ± 1.8
	T2	2.6 ± 1.6	2.4 ± 2.0	3.7 ± 1.6	2.8 ± 1.5
	T3	2.4 ± 1.3	2.5 ± 1.7	3.4 ± 1.7	2.5 ± 1.6
	T1–T0	− 1.5 (− 2.3, − 0.6)	− 0.9 (− 1.9, 0.1)	0.1 (− 0.4, 0.5)	− 0.3 (− 1.0, 0.3)
	T2–T0	− 1.6 (− 2.2, − 0.9)	− 0.8 (− 2.3, 0.6)	0.2 (− 0.6, 1.1)	− 0.8 (− 1.3, − 0.3)
	T3–T0	− 1.9 (− 2.7, − 1.0)	− 0.8 (− 1.8, 0.2)	− 0.1 (− 0.8, 0.7)	− 1.1 (− 1.8, − 0.4)
BP: Pain Interf Mean ± SD MD (95% CI)	T0	4.2 ± 2.1	3.3 ± 1.6	4.2 ± 2.2	3.4 ± 2.1
	T1	2.2 ± 1.0	2.3 ± 1.2	3.1 ± 2.0	3.2 ± 2.3
	T2	2.2 ± 1.6	2.6 ± 2.0	3.2 ± 1.8	2.7 ± 2.1
	T3	1.9 ± 1.0	2.5 ± 1.8	2.9 ± 1.7	2.2 ± 2.0
	T1–T0	− 2.0 (− 3.0, − 1.0)	− 1.0 (− 1.8, − 0.1)	− 1.1 (− 2.2, 0.0)	− 0.2 (− 0.8, 0.4)
	T2–T0	− 2.0 (− 2.8, − 1.2)	− 0.7 (− 1.6, 0.3)	− 1.1 (− 2.0, − 0.1)	− 0.7 (− 1.2, − 0.2)
	T3–T0	− 2.3 (− 3.5, − 1.2)	− 0.8 (− 2.0, 0.4)	− 1.3 (− 2.4, − 0.2)	− 1.1 (− 1.9, − 0.4)
BPI: Worst pain Mean ± SD MD (95% CI)	T0	5.7 ± 2.0	5.1 ± 2.2	5.1 ± 2.1	5.1 ± 2.3
	T1	3.5 ± 1.6	3.5 ± 2.2	4.9 ± 2.2	4.7 ± 3.0
	T2	3.7 ± 2.0	3.4 ± 2.4	4.8 ± 1.9	4.3 ± 2.6
	T3	3.7 ± 2.1	3.5 ± 2.1	4.6 ± 2.2	3.9 ± 2.6
	T1–T0	− 2.3 (− 3.5, − 1.0)	− 1.5 (− 2.8, − 0.2)	− 0.2 (− 1.1, 0.7)	− 0.4 (− 1.8, 1.0)
	T2–T0	− 2.1 (− 3.2, − 0.9)	− 1.7 (− 3.4, 0.1)	− 0.3 (− 1.4, 0.7)	− 0.8 (− 1.9, 0.3)
	T3–T0	− 2.1 (− 3.3, − 0.8)	− 1.5 (− 2.7, − 0.3)	− 0.5 (− 1.7, 0.6)	− 1.2 (− 2.6, 0.2)
BPI: Least pain Mean ± SD MD (95% CI)	T0	2.8 ± 1.6	1.5 ± 1.5	2.2 ± 1.1	2.1 ± 1.6
	T1	2.3 ± 2.0	1.3 ± 1.5	2.0 ± 1.2	2.0 ± 1.7
	T2	1.7 ± 1.3	1.6 ± 2.1	2.3 ± 1.5	1.7 ± 1.6
	T3	1.2 ± 1.3	1.5 ± 1.6	2.0 ± 1.7	1.5 ± 1.6
	T1–T0	− 0.5 (− 1.4, 0.4)	− 0.1 (− 0.8, 0.6)	− 0.2 (− 0.8, 0.4)	− 0.1 (− 0.5, 0.2)
	T2–T0	− 1.1 (− 1.7, − 0.4)	0.1 (− 1.1, 1.3)	0.1 (− 0.8, 0.9)	− 0.4 (− 0.9, 0.1)
	T3–T0	− 1.6 (− 2.4, − 0.8)	0.1 (− 0.9, 1.0)	− 0.2 (− 0.9, 0.5)	− 0.7 (− 1.2, − 0.1)
BPI: Average pain Mean ± SD MD (95% CI)	T0	4.3 ± 1.6	3.7 ± 1.7	3.3 ± 1.5	4.1 ± 1.8
	T1	2.8 ± 1.5	2.5 ± 1.5	3.7 ± 1.4	3.5 ± 2.1
	T2	3.0 ± 2.0	2.7 ± 2.1	3.9 ± 1.8	3.1 ± 1.7
	T3	2.5 ± 1.5	2.5 ± 1.7	3.5 ± 1.9	2.8 ± 2.3
	T1–T0	− 1.5 (− 2.2, − 0.7)	− 1.1 (− 2.2, 0.0)	0.3 (− 0.3, 0.9)	− 0.5 (− 1.3, 0.2)
	T2–T0	− 1.3 (− 2.0, − 0.6)	− 0.9 (− 2.4, 0.6)	0.5 (− 0.6, 1.7)	− 1.0 (− 1.7, − 0.3)
	T3–T0	− 1.7 (− 2.5, − 0.9)	− 1.1 (− 2.2, − 0.1)	0.2 (− 0.7, 1.1)	− 1.3 (− 2.1, − 0.4)
BPI_Current pain Mean ± SD MD (95% CI)	T0	3.8 ± 2.5	2.9 ± 2.3	3.0 ± 1.6	3.1 ± 1.8
	T1	2.3 ± 2.0	2.0 ± 1.5	3.3 ± 1.9	2.9 ± 1.9
	T2	1.9 ± 1.7	2.0 ± 1.9	3.7 ± 2.0	2.2 ± 1.5
	T3	1.9 ± 1.7	2.3 ± 2.0	3.3 ± 2.1	1.9 ± 1.5
	T1–T0	− 1.5 (− 2.8, − 0.3)	− 0.9 (− 2.2, 0.5)	0.3 (− 0.3, 0.8)	− 0.3 (− 1.2, 0.6)
	T2–T0	− 1.9 (− 2.9, − 0.8)	− 0.9 (− 2.6, 0.9)	0.7 (− 0.5, 1.9)	− 0.9 (− 1.8, − 0.1)
	T3–T0	− 1.9 (− 3.2, − 0.7)	− 0.6 (− 2.1, 0.9)	0.3 (− 0.5, 1.0)	− 1.2 (− 1.9, − 0.5)
RMDQ Mean ± SD MD (95% CI)	T0	10.7 ± 4.6	8.9 ± 3.2	10.6 ± 4.3	8.8 ± 4.6
	T1	6.9 ± 3.3	6.7 ± 2.8	8.4 ± 4.6	7.5 ± 3.9
	T2	7.1 ± 4.3	6.3 ± 3.3	8.3 ± 4.1	7.7 ± 4.3
	T3	6.1 ± 3.8	6.6 ± 4.2	7.4 ± 3.8	7.2 ± 4.0
	T1–T0	− 3.7 (− 5.8, − 1.7)	− 2.1 (− 4.0, − 0.2)	− 2.2 (− 4.4, 0.0)	− 1.3 (− 2.1, − 0.4)
	T2–T0	− 3.5 (− 5.4, − 1.7)	− 2.5 (− 4.3, − 0.7)	− 2.4 (− 4.4, − 0.4)	− 1.1 (− 1.7, − 0.4)
	T3–T0	− 4.5 (− 6.1, − 2.9)	− 2.3 (− 4.6, 0.0)	− 3.3 (− 5.5, − 1.0)	− 1.6 (− 2.5, − 0.7)

*BPI* Brief Pain Inventory, *CI* confidence interval, *dACC* dorsal anterior cingulate cortex, *Intf.* interference, *MD* mean difference, *pgACC* pregenual anterior cingulate cortex, *RMDQ* Roland Morris Disability Questionnaire, *SSC* somatosensory cortex, *SD* standard deviation, *T0* baseline, *T1* immediately post-treatment, *T2* 1 week follow up, *T3* 1 month follow up.

**Table 3:** Descriptive data for the secondary measures at all timepoints.

Variable	Time point	Group 1 (pgACC) (n = 15)	Group 2 (dACC + SSC) (n = 15)	Group 3 (Ratio) (n = 15)	Group 4 (Placebo) (n = 15)
MTS_Back Mean ± SD MD (95% CI)	T0	10.4 ± 15.9	15.6 ± 12.2	12.7 ± 12.7	9.7 ± 9.7
	T1	8.8 ± 10.9	12.3 ± 10.0	9.3 ± 9.0	7.6 ± 8.6
	T2	8.4 ± 11.3	9.3 ± 7.4	12.7 ± 9.8	8.5 ± 9.0
	T3	6.7 ± 5.5	10.8 ± 7.1	9.7 ± 8.8	7.4 ± 8.6
	T1–T0	− 1.6 (− 5.4, 2.1)	− 3.3 (− 8.6, 2.0)	− 3.3 (− 7.3, 0.6)	− 2.0 (− 4.8, 0.7)
	T2–T0	− 2.0 (− 5.6, 1.6)	− 6.4 (− 13.1, 0.4)	0.1 (− 4.1, 4.2)	− 1.2 (− 4.1, 1.7)
	T3–T0	− 3.8 (− 11.3, 3.8)	− 4.8 (− 10.7, 1.1)	− 2.9 (− 7.0, 1.1)	− 2.3 (− 5.0, 0.4)
MTS_Wrist Mean ± SD MD (95% CI)	T0	6.5 ± 12.5	6.7 ± 10.1	4.2 ± 7.0	4.6 ± 6.2
	T1	3.4 ± 5.0	5.7 ± 7.6	4.3 ± 5.6	4.7 ± 7.4
	T2	3.4 ± 4.0	5.0 ± 4.9	6.6 ± 7.7	5.7 ± 9.8
	T3	2.5 ± 3.5	4.3 ± 4.3	3.6 ± 4.9	4.2 ± 6.1
	T1–T0	− 3.1 (− 7.6, 1.4)	− 1.0 (− 6.2, 4.2)	0.1 (− 2.6, 2.8)	0.1 (− 2.1, 2.3)
	T2–T0	− 3.0 (− 9.1, 3.0)	− 1.7 (− 7.7, 4.3)	2.4 (0.3, 4.4)	1.1 (− 2.7, 4.9)
	T3–T0	− 4.0 (− 10.8, 2.8)	− 2.4 (− 7.3, 2.5)	− 0.6 (− 2.8, 1.5)	− 0.4 (− 1.8, 1.1)
PPT_Back Mean ± SD MD (95% CI)	T0	176.4 ± 118.8	228.6 ± 150.2	223.9 ± 158.2	342.6 ± 260.9
	T1	241.0 ± 203.6	267.9 ± 182.4	312.4 ± 195.1	351.3 ± 252.5
	T2	253.5 ± 212.2	285.5 ± 194.9	288.9 ± 161.9	362.9 ± 223.1
	T3	220.7 ± 193.5	279.3 ± 165.3	305.6 ± 166.7	357.9 ± 256.4
	T1–T0	64.7 (− 36.9, 166.2)	39.3 (− 2.8, 81.5)	88.4 (28.7, 148.1)	8.8 (− 70.6, 88.2)
	T2–T0	77.2 (− 23.8, 178.1)	56.9 (14.4, 99.3)	65.0 (21.5, 108.4)	20.3 (− 41.1, 81.7)
	T3–T0	44.3 (− 68.0, 156.6)	50.7 (− 2.6, 103.9)	81.7 (36.9, 126.4)	15.4 (− 99.5, 130.2)
PPT_Wrist Mean ± SD MD (95% CI)	T0	207.7 ± 137.5	236.7 ± 115.6	264.4 ± 228.8	265.9 ± 169.0
	T1	254.2 ± 181.5	227.2 ± 132.1	286.8 ± 230.1	298.7 ± 162.6
	T2	221.7 ± 111.8	242.9 ± 127.3	277.2 ± 184.6	308.4 ± 170.8
	T3	224.5 ± 123.9	239.7 ± 117.4	296.4 ± 213.4	309.1 ± 178.5
	T1–T0	46.5 (− 39.2, 132.2)	− 9.4 (− 35.5, 16.7)	22.4 (− 31.9, 76.6)	32.7 (− 28.7, 94.2)
	T2–T0	14.0 (− 63.9, 92.0)	6.3 (− 13.4, 26.0)	12.7 (− 38.3, 63.8)	42.4 (− 8.1, 93.0)
	T3–T0	16.8 (− 78.2, 111.7)	3.0 (− 21.7, 27.7)	32.0 (− 38.5, 102.5)	43.2 (− 12.0, 98.4)
CPM_30s Mean ± SD MD (95% CI)	T0	25.2 ± 35.9	30.8 ± 27.9	27.0 ± 32.0	31.3 ± 37.6
	T1	19.4 ± 34.5	13.2 ± 26.4	18.2 ± 24.0	24.1 ± 35.2
	T2	19.6 ± 25.8	23.3 ± 28.9	12.2 ± 21.5	9.3 ± 45.1
	T3	5.6 ± 26.5	18.0 ± 24.7	11.0 ± 15.1	22.9 ± 32.3
	T1–T0	− 5.8 (− 29.9, 17.7)	− 17.6 (− 33.7, − 1.4)	− 8.8 (− 34.4, 16.8)	− 7.3 (− 27.5, 12.9)
	T2–T0	− 5.6 (− 27.1, 15.9)	− 7.5 (− 19.9, 5.0)	− 14.8 (− 36.6, 7.0)	− 22.0 (− 47.8, 3.7)
	T3–T0	− 19.5 (− 40.6, 1.5)	− 12.8 (− 24.2, − 1.3)	− 16.0 (− 32.4, 0.4)	− 8.4 (− 21.0, 4.1)
CPM_60s Mean ± SD MD (95% CI)	T0	24.7 ± 35.0	30.7 ± 44.8	16.0 ± 47.0	10.5 ± 40.4
	T1	17.2 ± 24.6	8.9 ± 30.5	12.0 ± 22.8	4.9 ± 41.1
	T2	15.1 ± 22.9	3.8 ± 41.3	3.8 ± 29.4	− 2.7 ± 46.7
	T3	3.2 ± 22.7	20.2 ± 29.9	− 9.4 ± 33.7	4.4 ± 38.2
	T1–T0	− 7.5 (− 27.1, 12.0)	− 21.8 (− 43.8, 0.1)	− 4.1 (− 29.7, 21.6)	− 5.6 (− 24.9, 13.7)
	T2–T0	− 9.7 (− 30.0, 10.6)	− 27.0(− 51.0, − 2.9)	− 12.2 (− 49.6, 25.1)	− 13.2 (− 36.4, 10.0)
	T3–T0	− 21.5 (− 40.8, − 2.2)	− 10.5 (− 24.9, 3.9)	− 25.5 (− 49.0, − 2.0)	− 6.1 (− 14.7, 2.5)
CPM_90s Mean ± SD MD (95% CI)	T0	19.5 ± 26.9	22.7 ± 35.7	7.8 ± 46.7	13.2 ± 24.4
	T1	12.8 ± 32.1	11.8 ± 31.8	9.6 ± 25.8	7.0 ± 29.1
	T2	11.6 ± 38.7	10.4 ± 25.5	− 0.5 ± 25.6	5.7 ± 21.0
	T3	− 0.3 ± 17.7	12.7 ± 33.3	0.0 ± 36.8	7.4 ± 24.1
	T1–T0	− 6.7 (− 28.9, 15.5)	− 10.9 (− 28.7, 6.9)	1.8 (− 24.1, 27.7)	− 6.2 (− 25.5, 13.2)
	T2–T0	− 7.9 (− 31.9, 16.0)	− 12.3 (− 30.6, 5.9)	− 8.4 (− 40.5, 23.7)	− 7.5 (− 20.3, 5.3)
	T3–T0	− 19.8 (− 37.5, − 2.1)	− 10.0 (− 23.6, 3.5)	− 7.9 (− 28.6, 12.8)	− 5.8 (− 20.3, 8.6)
Pain Unpleasantness Mean ± SD MD (95% CI)	T0	4.7 ± 2.3	3.8 ± 1.2	4.1 ± 1.8	4.1 ± 2.4
	T1	3.0 ± 2.0	2.8 ± 1.3	4.2 ± 2.3	4.3 ± 2.6
	T2	3.6 ± 2.1	3.1 ± 2.4	4.7 ± 2.1	3.4 ± 2.7
	T3	2.9 ± 1.3	3.2 ± 1.9	4.1 ± 2.3	3.3 ± 2.7
	T1–T0	− 1.7 (− 2.7, − 0.6)	− 1.0 (− 1.8, − 0.2)	0.1 (− 1.2, 1.3)	0.1 (− 0.8, 1.0)
	T2–T0	− 1.1 (− 2.1, − 0.1)	− 0.7 (− 2.0, 0.7)	0.6 (− 0.5, 1.7)	− 0.7 (− 1.6, 0.2)
	T3–T0	− 1.7 (− 2.8, − 0.6)	− 0.6 (− 1.6, 0.4)	− 0.1 (− 1.2, 1.1)	− 0.9 (− 2.1, 0.4)
Pain Bothersomeness Mean ± SD MD (95% CI)	T0	4.9 ± 2.4	4.1 ± 1.9	4.3 ± 2.2	4.0 ± 2.4
	T1	2.7 ± 2.0	2.7 ± 1.9	4.1 ± 2.4	3.5 ± 2.1
	T2	3.1 ± 2.1	2.8 ± 2.3	4.2 ± 2.4	3.0 ± 2.1
	T3	2.7 ± 1.4	3.4 ± 2.3	4.1 ± 2.4	2.8 ± 2.1
	T1–T0	− 2.3 (− 3.7, − 0.8)	− 1.4 (− 2.3, − 0.5)	− 0.1 (− 1.5, 1.2)	− 0.5 (− 1.3, 0.4)
	T2–T0	− 1.9 (− 3.1, − 0.6)	− 1.3 (− 2.4, − 0.1)	− 0.1 (− 1.6, 1.4)	− 1.0 (− 1.7, − 0.3)
	T3–T0	− 2.3 (− 3.5, − 1.0)	− 0.7 (− 1.7, 0.4)	− 0.1 (− 1.5, 1.2)	− 1.2 (− 2.2, − 0.2)

*CI* confidence interval, *CPM* conditioned pain modulation, *dACC* dorsal anterior cingulate cortex, *MD* mean difference, *MTS* mechanical temporal summation, *pgACC* pregenual anterior cingulate cortex, *PPT* pressure pain threshold, *SSC* somatosensory cortex, *SD* standard deviation, *T0* baseline, *T1* immediately post-treatment, *T2* 1 week follow up, *T3* 1 month follow up.

#### Pain

All treatment groups demonstrated a favourable change in pain measures at all timepoints (Table 2, Fig. 5). However, comparatively the pgACC group demonstrated clinically meaningful trends of reduction (MD ≥ − 2) in pain (Table 2). At 1-month follow-up, the proportion of participants that demonstrated a meaningful reduction (MCID > 30%) for both pain severity and pain interference was higher in pgACC group compared to other treatment groups (Fig. 5).

#### Function

The RMDQ scores demonstrated a trend of reduction in disability in all treatment groups (Table 2, Fig. 5), with pgACC group exhibiting the highest decline in disability comparatively at all time points. At 1-month follow-up, the proportion of participants that demonstrated a meaningful reduction (MCID > 30%) in disability was greater in ISF-NF treatment groups [pgACC (73%), dACC + SSC (40%), and Ratio (47%)] when compared to Sham (20%) group (Fig. 5f).

#### Global perceived effect

At 1-month follow-up, the proportion of participants who perceived meaningful (≥ + 2) global effect was higher in pgACC (67%) and dACC + SSC (64%) group, when compared to ratio (46%) and sham (46%) group.

#### QST

Due to high variability in measures, no differences in trends were observed in any of QST variables at 1-month follow-up (Table 3).

### EEG measures

**Figure 6:**
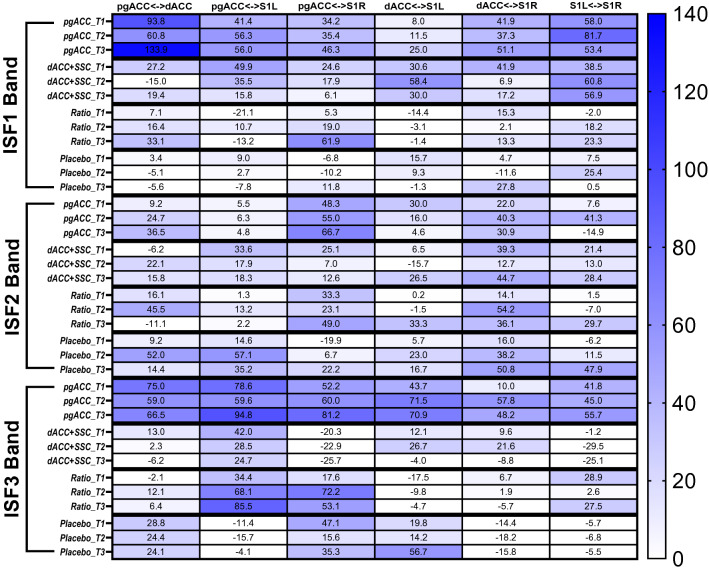
Heatmaps for mean percentage change to baseline in functional connectivity in the infraslow frequency bands. pgACC: pregenual anterior cingulate cortex, dACC: dorsal anterior cingulate cortex, SSC: primary somatosensory cortex, ISF1: Infraslow frequency- low band (0.01–0.04 Hz), ISF2: Infraslow frequency- mid band (0.05–0.07 Hz), ISF3: Infraslow frequency- high band (0.08–0.10 Hz), S1L: Primary Somatosensory cortex left, S1R: Primary Somatosensory cortex right, T0: baseline, T1: immediately post-treatment, T2: 1 week follow up, T3: 1 month follow up, <->: functional Connectivity between regions. Higher values represent increase in the functional connectivity compared to baseline.

Descriptive data for CD and FC measures at all time points are presented in Supplementary Tables S2 and S3 online. The CD values at the targeted brain regions (pgACC, dACC, and SSC), were comparable across treatment groups, with no changes observed across all time points. The FC measures demonstrated trend toward increased connectivity between the targeted brain regions in the treatment groups when compared to the Placebo groups. Figure 6 presents the heat map for mean percentage changes to baseline in FC between the targeted brain regions (pgACC, dACC, and SSC). At 1-month follow-up, the pgACC group demonstrated the highest magnitude of increases in FC, particularly in ISF1 and ISF3 bands.

## Discussion

Re-training cortical activity through real-time EEG-based source localised ISF-NF is a novel approach for treatment of chronic pain^43^. The use of NF training to treat various chronic conditions has attracted significant interest over the past decade. However, recent systematic reviews highlight that the evidence for effect of NF treatment for chronic pain, although promising, is of low quality, largely based on case-series and non-randomised studies^15,16^. Further, to date, only one open-label study has explored the EEG-based NF training for treatment of CLBP^18^. To our knowledge, this pilot study is the first double-blinded randomised placebo-controlled feasibility trial to explore source-localised EEG ISG-NF training for treatment of CLBP. This study primarily explored feasibility, safety, and acceptability of EEG-based NF training for CLBP, as these are important domains in developing any health care interventions^54^.

Our results demonstrate that a fully powered trial to test efficacy of EEG-based ISF-NF training for treatment of CLBP is feasible. A sizable number of individuals with CLBP were interested and willing to participate in EEG-NF intervention. Our recruitment rate was comparable to other CLBP intervention studies^55^, and our randomization rate was 100%. Participant retention rates (75%) and treatment adherence (~ 80%) was high. All participants also seemed to have consistently maintained moderate to high levels of mood, motivation, and engagement with the ISF-NF training throughout the treatment period, which are recognised as important predictors influencing treatment outcomes^56^. Our study was also able to successfully blind participants and outcome assessor to treatment groups.

Study findings also confirms safety of EEG-based ISF-NF training for treatment of CLBP. Our study used an extensive DESS scale^44^ administered before and after each training session to assess immediate and delayed side effects of ISF-NF training. No serious adverse events were reported by any participant. Side effects reported were mild, transient, and self-resolved post-training. These findings are consistent with previous NF studies using other training protocols for chronic pain^15,17^, and in studies using ISF-NF protocol for food addiction^26^. Increased dreaming was a common side effect reported by ISF-NF training groups. Participants reported increases in quantity of dreaming, with more vivid dreams. Increased dreaming following NF sessions has been previously reported and are a positive indication of procedural, nondeclarative learning that occurs during training^57^. Further, following a NF session, vivid dreaming has also been suggested to serve as an early marker of brain’s response to training^57^. Interesting, none of the participants reported increased dreaming in Placebo-NF group, which might further indicate specificity of brain learning in ISF-NF groups.

Although treatment was acceptable and participants were moderate-to-highly satisfied, some important recommendations were made. A few participants indicated that they were unsure of mental strategies to use and would have benefited from some examples. While some studies^58^ indicate that contingent feedback without explicit mental strategies enables more effective learning, others studies^59,60^ indicate its use improves treatment outcomes compared to no strategy. In line with dual-process and multistage theories of NF learning^14,61–63^, our study let participants explore and try several approaches to infer whether a specific mental strategy influenced their response. Future research could explore the effect of no/specific mental strategies in moderating treatment outcomes. Some participants reported inconvenience due to gel application and re-scheduled their training sessions to after-work hours. Incorporation of modern dry EEG electrode system for administering NF might likely improve its acceptability and interest. While a few studies^64,65^ claim accuracy of dry EEG electrode system as comparable to wet electrodes, these have been reported primarily for recording of higher frequency bands. Future research is needed to assess the accuracy of a dry EEG electrode system for recording ISF bands. Other concerns included difficulty sitting still for total treatment duration (30 min). We asked participants to sit still as movement artefacts could produce sounds, and those artefact sounds could be misinterpreted as real feedback. Recent advances of wireless EEG^64,66^ that are less sensitive to motion artefacts and frequent breaks during the training session or interval training methods might improve participant’s experiences and comfort during training.

Exploratory findings on clinical measures demonstrated a decreasing trend in pain and disability in all treatment groups. Our results are comparable to the previous NF studies in chronic pain conditions, who also demonstrated significant reductions in pain and disability following training^15–17^. However, it is important to emphasize that our pilot study was a feasibility trial and thus only descriptively compared the between-group trend of effect, and did not have power to statistically examine the group X time effects on outcomes. Thus, a fully powered trial is required before drawing any definitive conclusions based on this study observations.

Our pilot study results showed that ISF-NF treatment targeting pgACC had the highest proportion of participants who exhibited sustained clinically meaningful decrease in both pain and disability and had improved global perceived effect at 1-month follow-up. EEG FC measures also demonstrated highest magnitude of changes in the pgACC group, demonstrating the specificity of EEG-NF training. Further, in a recent secondary analysis of our data, we also demonstrate that the ISF-NF uptraining the pgACC increases the effective connectivity from the pgACC to SSC and this is correlated with greater reductions in pain severity^67^. These findings support the notion that chronic pain likely results from a deficiency of pain inhibition and NF protocols that strengthens the effective connectivity from the pgACC to SSC may be optimal for pain suppression. Furthermore, while recent evidence^68^ raises doubts concerning the proposed mechanism of action behind the behavioural effects of neurofeedback, attributing treatment effects to non-specific factors (such as treatment expectations, motivation, awareness, attention, and engagement), all our study groups were highly comparable at baseline (Table 1), which additionally demonstrates the specific effects of the EEG-NF training on clinical outcomes.

The greatest clinical effects demonstrated in the pgACC uptraining group could also be attributed to the simplicity of the protocol and ease in learning (i.e., uptraining CD of a single brain region). Previous NF studies in chronic pain conditions have demonstrated that complex training protocols (e.g., adding beta down training to the protocol of uptraining of sensorimotor rhythm and down training of theta) reduced training effectiveness, and increasing the number of training sessions increased pain reduction for such protocols^15^. These findings suggest that training multiple cortical regions and more complex protocols (e.g., simultaneously uptraining and down training different brain regions) might require an increased number of training sessions. Further, a step-by-step or phased systematic and individualised training approach (e.g., uptraining pgACC, followed by downtraining dACC + SSC, and finally training to increase the ratio) may be easier to learn and might improve treatment outcomes to a greater effect.

We also observed a time course effect in several clinical measures, where the pain and function continued to improve over time. These findings have also been observed previously^69,70^, where clinical symptoms and neurophysiological variables neither regressed to baseline nor remained stable but continued to improve for weeks following NF training. While this might be due to practice effects of learning to control neural activity or reflect slow consolidation processes^71,72^, other mechanistic speculations such as self-reinforcement of brain over time to strengthen the correlational structure of network brain activity have also been proposed^69^ and need further research. Based on these findings, it is recommended that future studies should include a longer follow-up period to sample the time point of greatest effect^69^.

### Limitations

The primary limitations of this pilot feasibility study were small sample size and descriptive comparisons to infer trends in clinical and EEG outcomes. However, these limitations reflect the purpose of our feasibility study, which was to provide estimates of clinical outcome measures (pain and disability) to support sample size calculation for use in fully powered trial. Based on results of this study, a future fully powered trial will be conducted to evaluate efficacy of ISF-NF training. The future RCT could tests the effect modifiers of LBP subgroups on clinical outcomes. Another limitation is that the XYZ coordinates for the 3 selected areas were based on a neurosynth meta-analysis of pain. While this is should be optimal for the group, this may be suboptimal for the individual. For example, the selection of the pgACC XYZ coordinates was based on the neurosynth meta-analysis for pain, yet there may may be more optimal ROIs to target for the individual patient. Similarly, the connectivity between the SSC and salience network is topographic, i.e. based on which part of the body is in pain^11^, and thus training the entire SSC may be suboptimal, in comparison to only training the XYZ coordinates that somatotopically relate to the painful body area.

## Conclusions

The ISF-NF training is feasible, safe, and an acceptable treatment approach for CLBP. A positive trend of the effect of ISF-NF treatment was observed on pain and disability outcomes in all groups. In particular, the pgACC uptraining group experienced favourable outcomes and perceived the intervention to be highly effective. However, a fully powered RCT is needed to evaluate the clinical efficacy of ISF-NF training in people with CLBP.

## Supplementary Information

Supplementary Information.

## Data Availability

Datasets generated during and/or analysed during the current study are available from the corresponding author upon reasonable request.
