# Supplementary material for: Exploring electroencephalographic infraslow neurofeedback treatment for chronic low back pain: a double-blinded safety and feasibility randomized placebo-controlled trial
Source: Sci Rep. 2023 Jan 20;13:1177. doi: 10.1038/s41598-023-28344-2 (PMC9860016; doi:10.1038/s41598-023-28344-2)
Supplement: Supplementary file 1 — Supplementary Information. [file 41598_2023_28344_MOESM1_ESM.pdf]

Supplementary Table S1: Qualitative feedback Summary

Theme	Feedback	Sample feedback quotes
**Time Commitment**	Although a few participants felt that the study was a huge commitment over a long period of time, others felt that it was easy to fit in their schedule and were positive about it.	“Quite a commitment but with prospect of reducing pain manageable.”<br>“Was a huge commitment timewise, over a long period of time.”<br>“It takes a lot of time, but I feel like I did get results.”<br>“The time used for it and the frequency made it hard to balance other aspects of the week's during the training.”<br>“I feel like a treatment was not very long or invasive and very easy to fit into my schedule. Therefore, even a slight decrease and back pain is worth the time and effort.”
**Improved knowledge**	Participants felt that participating in the study has improved their knowledge about neurofeedback, pain perception, and the role of brain in pain.	“Interesting learning about how neurofeedback works and how it can be used for pain.”<br>“It has been very interesting. Learning different ways to concentrate and learning about how neurofeedback works.”<br>“Yes, a clearer understanding of how the mind may control some of the pain. Comfort is knowing there is a treatment that can help.”<br>“It's been interesting in terms of how complex our brain is in the ways we are able to see how we respond to pain.”<br>“Yes, the brain is much more complex than I thought it was because before the treatment phase I didn't realise the brain could be trained to respond differently to pain.”<br>“I've never heard about neurofeedback treatment before, but now I know there is a treatment like that.”
**Enjoyment**	Participants reported that the neurofeedback experience was very interesting/ fascinating, and they enjoyed the training sessions.	“It was just really fascinating trying to figure out which parts of the brain were involved in which tones. I could have done that many other times as I really wanted to play with that instrument more. Thoroughly enjoyable.”<br>“A very cool learning experience. It's a type of treatment I didn't expect to receive but I'm surprised about how effective it's been for me, and I enjoyed being part of it.”

Theme	Feedback	Sample feedback quotes
		"Making the sounds with my brain waves and changing the frequencies of them has been incredibly fascinating. I would love to do that part of it more. At that start I really enjoyed trying to play a tune to the tester. After that to focus on making different tones, especially at higher pitches was really fun. All in all I really enjoyed the training and would love to do more."<br>"Enjoyable. Entire process has been well-organised and easy to participate in."<br>"I felt excited and enjoyed being part of this study…"<br>"I found it interesting and very helpful for a positive result by the end. I have been happy and motivated to attend."
**Study staff**	A few participants commented that the study staff were nice, kind, friendly and dedicated.	"Orchestrators of the treatment and follow-up sessions are amazing, super kind and informative. Really enjoyed meeting them."<br>"I've met some nice people who take their work seriously.<br>"The staff are very hospitable and nice."<br>"Entire process has been well-organised and easy to participate in."<br>"Friendly people, professional attitude."
**Treatment Position and duration**	A few participants found it hard to sit still and felt that the 30minutes treatment duration was too long to concentrate and be awake.	"I think 30min is too long to focus on sound. It's easy to lose concentration."<br>"Back did get a little sore at times when trying to stay still for 30 mins."<br>"Not easy to keep awake or not fall to sleep in the middle of the period."<br>"That was interesting coming to terms with sitting still for 30 minutes. I wouldn't have thought I could do that due to my pain."<br>"Challenging trying to stay still and not move. At times challenging to stay awake during sessions."<br>"Sitting still for 30 minutes is harder than I thought."
**Mental strategy**	Some participants were not sure of the mental strategies to use and	"Maybe some examples of potential mental techniques, I wasn't entirely sure what I was meant to do at times."

Theme	Feedback	Sample feedback quotes
	suggested to have provided some examples.	"Better instruction as to what to think about during the session or what to concentrate on."<br>"A suggestion would be to potentially teach patients mental strategies to stay focused on the feedback played as during the early sessions. I found it different to stay engaged in the training."
**Treatment effects**	Several participants reported a positive effect of neurofeedback treatment not only on their pain, but also in their psychological symptoms and other non-specific symptoms. Further they reported improved activity levels, social participation, quality of life and well being	"I'm more active and alert and don't need to rest during the day anymore! I can carry my grandson without any/much difficulty now. And I can garden!"<br>"Yes, it has noticeably improved my back pain. Seems to have improved some of my other symptoms too."<br>"It was actually really relaxing and stress relieving. I found my anxiety issues were greatly improved the week's doing it and have actually continued at the reduced level."<br>"First time, I had the best sleep in a very long time, so I think that is a benefit."<br>"I also learned 3 x 30 minutes of quiet contemplative alone time improves anxiety symptoms massively."<br>"I still get a sore back lifting etcetera, but I find some general things like driving and walking in the morning. I feel it has improved."<br>"It also helps my urge to pee and urge incontinence for the better."<br>"I have learnt to pay more attention on things that really matter and focus on the present and the things I have to do now, while still caring for the future. Yes, I feel less pain and can snap out of it easier."<br>"Yes, my back pain is still there but I feel I don't focus as much on it. So, it doesn't bother me as much."<br>"I feel I have gained being more active and positive helps my back pain. Also, it has improved the quality of my daily life in my mood."<br>"It was interesting! Pain still there but is in sleep mode! Not annoying anymore."<br>"Uncertain if NF helped with physical pain but I think it may have helped to cope with emotional distress/dysregulation."<br>"Might have improved my well-being. It has gone up over this. Unsure why."<br>"I've gained a better day-to-day quality of life and a good learning experience on meditation and focus."

Theme	Feedback	Sample feedback quotes
		“Should definitely try this as a migraine treatment. Worked wonders. Has also massively improved my stress management abilities.”
**No of sessions**	A few participants reported ongoing benefit and suggested that more training sessions would help enhance their learning.	“Wish it could go longer to have ongoing benefit.”<br>“I would love more training as I felt it is an excellent way to learn how to meditate deeper in those part of the brain and I feel I was only just starting to really get into a state deep enough to really make the noises happen properly (ie for a long time and smoothly). More time on training would help to learn to keep making the noises and at a higher pitch.”<br>“I would possibly need more sessions to benefit.”
**Equipment and testing environment**	Some participants reported inconvenience due to the EEG cap, gel use, speakers, and the furniture, and suggested changes to improve comfort and acceptability of the treatment.	“Wearing the headgear can often become painful in my neck, shoulders and jaw. It could be difficult to relax and fully embrace the treatment.”<br>“EEG cap has been quite claustrophobic; it's been hard to combat that.”<br>“For future if treatment is successful, I recommend switching headphones instead of speakers.”<br>“Minimise background noise, Warning about gel use! Wasn’t expecting that it might put women off messing up hair.”<br>“Only the Velcro on the cap if this was silicon it would not be so scratchy. Also, the chair, is there a higher backed chair that could be used as the tension in your shoulders gets quite high and making conscience relaxation suggestions to your body to stop stiffening up and stay relaxed.”<br>“Strap around the neck is uncomfortable - padding would help.”<br>“Gel was inconvenient. Needed to coordinate hair washing days.”<br>“Background sounds are distracting and make it hard. Maybe using headphones or something so you are literally in your own world.”
**Worthiness**	Several participants felt that the neurofeedback treatment was worth the time and effort, and that	“Neuro feedback is definitely worth a try as it has helped me.”<br>“Give it a go! Can only stay the same at worst or give improvement.”<br>“I would recommend to everybody.”

Theme	Feedback	Sample feedback quotes
	they would recommend it to others.	“Definitely worth a go, regardless of the treatment category you end up in. It's worth learning and advancing more in this type of medical science and you may very well benefit from it.”<br>“I would highly recommend this interventional treatment. My experiences that it is worthwhile and effective.”<br>“It was really worth the time and effort. It kept me positive and active.”<br>“Entirely worthwhile, mostly due to the perception that my back pain has been aided by these treatment sessions. Whether or not the treatment worked (it seems to have) these sessions were worth my time.”

Supplementary Table S2. Descriptive data for the EEG log-transformed current density changes at all timepoints

Variable	Time point	Group 1 (pgACC)	Group 2 (dACC+SSC)	Group 3 (Ratio)	Group 4 (Placebo)
**pgACC_ISF1**	T0	12.5 ± 1.0	11.2 ± 1.1	11.1 ± 1.0	10.9 ± 0.9
Mean ± SD	T1	12.1 ± 1.0	10.9 ± 1.2	11.9 ± 0.8	11.0 ± 0.6
	T2	12.2 ± 1.1	10.8 ± 1.5	11.7 ± 1.0	10.9 ± 1.0
	T3	11.8 ± 1.1	11.1 ± 1.6	11.6 ± 1.0	10.6 ± 0.6
MD (95% CI)	T1-T0	-0.5 (-1.3, 0.4)	-0.3 (-0.9, 0.2)	0.8 (0.1, 1.4)	0.1 (-0.4, 0.6)
	T2-T0	-0.3 (-1.0, 0.4)	-0.4 (-0.9, 0.1)	0.7 (-0.1, 1.4)	-0.1 (-0.8, 0.7)
	T3-T0	-0.7 (-1.4, 0.0)	-0.1 (-0.8, 0.6)	0.5 (-0.3, 1.4)	-0.3 (-0.9, 0.3)
**pgACC_ISF2**	T0	10.8 ± 0.9	9.8 ± 1.1	9.7 ± 1.3	9.8 ± 1.0
Mean ± SD	T1	10.6 ± 1.0	9.3 ± 1.1	10.5 ± 0.9	9.8 ± 0.8
	T2	10.7 ± 1.1	9.4 ± 1.3	10.3 ± 1.1	9.6 ± 1.0
	T3	10.3 ± 1.2	9.4 ± 1.4	10.0 ± 1.0	9.3 ± 0.7
MD (95% CI)	T1-T0	-0.2 (-0.9, 0.5)	-0.5 (-0.8, 0.1)	0.7 (0.1, 1.4)	0.0 (-0.5, 0.5)
	T2-T0	-0.1 (-0.6, 0.4)	-0.4 (-0.9, 0.2)	0.5 (-0.3, 1.3)	-0.2 (-0.9, 0.6)
	T3-T0	-0.5 (-1.2, 0.1)	-0.4 (-1.0, 0.2)	0.3 (-0.6, 1.2)	-0.4 (-1.1, 0.3)
**pgACC_ISF3**	T0	10.1 ± 0.9	9.2 ± 1.0	9.0 ± 1.2	9.0 ± 1.1
Mean ± SD	T1	9.9 ± 1.0	8.6 ± 1.0	9.7 ± 0.9	9.0 ± 0.8
	T2	9.9 ± 1.0	8.7 ± 1.3	9.5 ± 1.1	8.7 ± 1.0
	T3	9.6 ± 1.2	8.7 ± 1.4	9.3 ± 0.9	8.7 ± 0.9
MD (95% CI)	T1-T0	-0.3 (-1.0, 0.5)	-0.6 (-1.0, -0.2)	0.6 (-0.1, 1.3)	0.0 (-0.5, 0.5)
	T2-T0	-0.2 (-0.8, 0.3)	-0.5 (-1.0, 0.1)	0.4 (-0.3, 1.2)	-0.3 (-1.0, 0.5)
	T3-T0	-0.6 (-1.2, 0.1)	-0.4 (-1.0, 0.2)	0.3 (-0.5, 1.0)	-0.3 (-1.0, 0.3)
**dACC_ISF1**	T0	12.1 ± 1.2	11.0 ± 1.3	11.3 ± 1.3	11.1 ± 0.6
Mean ± SD	T1	11.9 ± 1.3	11.0 ± 1.2	11.6 ± 1.1	10.7 ± 0.6
	T2	11.5 ± 1.4	11.0 ± 1.1	11.1 ± 0.9	10.9 ± 0.9
	T3	11.4 ± 1.2	11.0 ± 1.3	11.0 ± 0.5	10.8 ± 1.1
MD (95% CI)	T1-T0	-0.2 (-1.0, 0.5)	0.0 (-0.5, 0.5)	0.3 (-0.4, 0.9)	-0.3 (-0.9, 0.2)
	T2-T0	-0.5 (-1.3, 0.2)	0.0 (-0.6, 0.5)	-0.2 (-1.0, 0.5)	-0.2 (-0.6, 0.2)
	T3-T0	-0.7 (-1.4, 0.0)	0.0 (-0.7, 0.6)	-0.4 (-1.1, 0.3)	-0.2 (-0.8, 0.4)
**dACC_ISF2**	T0	10.3 ± 1.0	9.5 ± 1.4	10.0 ± 1.5	9.8 ± 0.7
Mean ± SD	T1	10.3 ± 1.2	9.3 ± 1.1	10.2 ± 1.3	9.4 ± 0.7
	T2	10.0 ± 1.4	9.2 ± 1.1	9.6 ± 1.0	9.6 ± 0.9
	T3	9.8 ± 1.2	9.4 ± 1.3	9.5 ± 0.7	9.6 ± 1.3
MD (95% CI)	T1-T0	0.0 (-0.6, 0.5)	-0.2 (-0.8, 0.4)	0.3 (-0.4, 0.9)	-0.4 (-0.9, 0.1)
	T2-T0	-0.3 (-0.8, 0.3)	-0.3 (-1.0, 0.4)	-0.4 (-1.3, 0.5)	-0.3 (-0.6, 0.1)
	T3-T0	-0.5 (-1.1, 0.1)	-0.1 (-0.9, 0.6)	-0.5 (-1.3, 0.4)	-0.3 (-0.9, 0.4)
**dACC_ISF3**	T0	9.5 ± 1.0	8.8 ± 1.4	9.4 ± 1.5	9.1 ± 0.8
Mean ± SD	T1	9.4 ± 1.2	8.5 ± 1.1	9.5 ± 1.4	8.6 ± 0.7
	T2	9.2 ± 1.3	8.5 ± 1.0	8.8 ± 1.0	8.6 ± 0.9
	T3	9.2 ± 1.2	8.6 ± 1.2	8.8 ± 0.8	8.7 ± 1.4
MD (95% CI)	T1-T0	-0.1 (-0.6, 0.4)	-0.3 (-0.9, 0.3)	0.1 (-0.6, 0.8)	-0.5 (-1.1, 0.0)
	T2-T0	-0.4 (-1.0, 0.2)	-0.2 (-1.0, 0.5)	-0.6 (-1.6, 0.4)	-0.5 (-0.9, 0.2)
	T3-T0	-0.4 (-1.0, 0.3)	-0.1 (-0.9, 0.6)	-0.6 (-1.4, 0.2)	-0.4 (-1.1, 0.3)
**S1L_ISF1**	T0	12.4 ± 1.0	11.4 ± 1.2	11.9 ± 1.5	11.3 ± 0.7
Mean ± SD	T1	12.6 ± 1.4	11.1 ± 1.0	12.2 ± 1.3	11.3 ± 0.8
	T2	12.4 ± 1.5	11.3 ± 1.3	11.9 ± 1.2	11.1 ± 0.8
	T3	12.2 ± 1.4	11.6 ± 1.3	11.4 ± 0.8	11.1 ± 0.9
MD (95% CI)	T1-T0	0.2 (-0.6, 1.0)	-0.3 (-0.6, 0.1)	0.3 (-0.3, 0.8)	-0.1 (-0.5, 0.4)
	T2-T0	0.0 (-0.9, 0.9)	-0.1 (-0.8, 0.6)	-0.1 (-1.3, 1.2)	-0.2 (-0.6, 0.2)

Variable	Time point	Group 1 (pgACC)	Group 2 (dACC+SSC)	Group 3 (Ratio)	Group 4 (Placebo)
	T3-T0	-0.2 (-1.2, 0.8)	0.2 (-0.5, 1.0)	-0.5 (-1.5, 0.5)	-0.2 (-0.8, 0.3)
**S1L_ISF2**	T0	10.7 ± 1.1	9.8 ± 1.1	10.6 ± 1.8	10.0 ± 0.8
Mean ± SD	T1	10.8 ± 1.3	9.5 ± 1.0	10.7 ± 1.7	9.8 ± 1.0
	T2	10.7 ± 1.3	9.6 ± 1.2	10.3 ± 1.1	9.7 ± 0.9
	T3	10.5 ± 1.4	10.0 ± 1.3	10.0 ± 0.9	9.6 ± 1.1
MD (95% CI)	T1-T0	0.2 (-0.5, 0.8)	-0.3 (-0.8, 0.2)	0.1 (-0.5, 0.7)	-0.3 (-0.7, 0.2)
	T2-T0	0.0 (-0.6, 0.6)	-0.2 (-0.8, 0.5)	-0.3 (-1.6, 1.0)	-0.4 (-0.8, 0.1)
	T3-T0	-0.1 (-0.9, 0.6)	0.2 (-0.6, 1.0)	-0.6 (-1.6, 0.5)	-0.4 (-1.1, 0.3)
**S1L_ISF3**	T0	9.8 ± 1.2	9.0 ± 1.1	9.8 ± 1.7	9.2 ± 0.8
Mean ± SD	T1	10.1 ± 1.3	8.7 ± 1.0	9.9 ± 1.6	8.9 ± 1.1
	T2	9.7 ± 1.3	8.8 ± 1.2	9.6 ± 1.4	8.8 ± 1.0
	T3	9.7 ± 1.4	9.1 ± 1.3	9.1 ± 1.0	9.0 ± 1.2
MD (95% CI)	T1-T0	0.3 (-0.3, 0.9)	-0.3 (-0.8, 0.1)	0.1 (-0.5, 0.6)	-0.2 (-0.7, 0.2)
	T2-T0	-0.1 (-0.7, 0.6)	-0.3 (-1.0, 0.5)	-0.2 (-1.4, 1.0)	-0.4 (-0.8, 0.0)
	T3-T0	0.0 (-0.8, 0.8)	0.1 (-0.6, 0.8)	-0.7 (-1.7, 0.2)	-0.2 (-0.9, 0.5)
**S1R_ISF1**	T0	12.2 ± 1.4	11.3 ± 1.3	11.8 ± 1.4	11.4 ± 0.8
Mean ± SD	T1	12.0 ± 1.5	11.1 ± 1.2	11.9 ± 1.2	11.2 ± 0.7
	T2	12.0 ± 1.3	11.1 ± 1.2	11.6 ± 1.3	11.0 ± 1.1
	T3	12.0 ± 1.2	11.6 ± 1.4	11.4 ± 0.8	10.9 ± 1.1
MD (95% CI)	T1-T0	-0.2 (-0.9, 0.5)	-0.2 (-0.6, 0.3)	0.0 (-0.6, 0.6)	-0.3 (-0.8, 0.3)
	T2-T0	-0.3 (-1.0, 0.5)	-0.2 (-0.9, 0.5)	-0.2 (-1.4, 0.9)	-0.4 (-0.9, 0.0)
	T3-T0	-0.3 (-1.2, 0.6)	0.3 (-0.5, 1.1)	-0.5 (-1.5, 0.5)	-0.5 (-1.0, 0.0)
**S1R_ISF2**	T0	10.5 ± 1.4	9.8 ± 1.4	10.3 ± 1.4	10.0 ± 0.8
Mean ± SD	T1	10.4 ± 1.6	9.5 ± 1.0	10.4 ± 1.4	9.7 ± 0.8
	T2	10.3 ± 1.2	9.6 ± 1.1	10.1 ± 1.3	9.7 ± 1.0
	T3	10.3 ± 1.2	9.8 ± 1.2	9.7 ± 0.9	9.6 ± 1.2
MD (95% CI)	T1-T0	-0.1 (-0.6, 0.5)	-0.3 (-0.8, 0.2)	0.1 (-0.5, 0.7)	-0.2 (-0.8, 0.3)
	T2-T0	-0.2 (-0.9, 0.6)	-0.2 (-0.8, 0.5)	-0.2 (-1.3, 0.9)	-0.3 (-0.8, 0.2)
	T3-T0	-0.1 (-1.0, 0.8)	0.0 (-0.8, 0.9)	-0.6 (-1.5, 0.4)	-0.4 (-1.0, 0.2)
**S1R_ISF3**	T0	9.6 ± 1.3	9.2 ± 1.3	9.8 ± 1.4	9.3 ± 0.9
Mean ± SD	T1	9.5 ± 1.5	8.9 ± 1.1	9.6 ± 1.4	8.8 ± 0.9
	T2	9.6 ± 1.4	8.8 ± 1.3	9.1 ± 1.4	8.7 ± 1.1
	T3	9.5 ± 1.4	9.0 ± 1.2	9.0 ± 0.9	8.6 ± 1.3
MD (95% CI)	T1-T0	-0.1 (-0.6, 0.4)	-0.3 (-0.7, 0.1)	-0.1 (-0.7, 0.5)	-0.4 (-0.9, 0.0)
	T2-T0	0.0 (-0.6, 0.5)	-0.4 (-1.3, 0.4)	-0.7 (-1.7, 0.4)	-0.6 (-1.1, -0.1)
	T3-T0	-0.1 (-1.0, 0.9)	-0.3 (-1.1, 0.6)	-0.8 (-1.5, 0.1)	-0.6 (-1.2, -0.1)

CI: Confidence Interval, dACC: dorsal anterior cingulate cortex, ISF1: Infraslow frequency- low band, ISF2: Infraslow frequency- mid band, ISF3: Infraslow frequency- high band, MD: Mean Difference, pgACC: pregenual anterior cingulate cortex, SD: Standard Deviation, S1L: Primary Somatosensory cortex left, S1R: Primary Somatosensory cortex right, T0: Baseline, T1: Immediately post-treatment, T2: One week follow up, T3: One month follow up.

Supplementary Table S3. Descriptive data for the EEG lagged linear functional connectivity measures at all timepoints

Variable	Time point	Group 1 (pgACC)	Group 2 (dACC+SSC)	Group 3 (Ratio)	Group 4 (Placebo)
**pgACC<->dACC_ISF1**	T0	1.5 ± 0.8	1.6 ± 0.7	1.2 ± 0.3	1.7 ± 0.9
Mean ± SD	T1	2.0 ± 1.0	1.8 ± 0.4	1.3 ± 0.5	1.6 ± 1.0
	T2	1.8 ± 1.4	1.3 ± 0.5	1.3 ± 0.6	1.3 ± 0.6
	T3	2.5 ± 1.3	1.6 ± 0.8	1.5 ± 0.6	1.4 ± 0.6
MD (95% CI)	T1-T0	0.6 (-0.4, 1.5)	0.2 (-0.1, 0.5)	0.1 (-0.2, 0.3)	-0.1 (-0.5, 0.4)
	T2-T0	0.3 (-0.7, 1.4)	-0.3 (-0.7, 0.1)	0.2 (-0.2, 0.5)	-0.4 (-1.1, 0.4)
	T3-T0	1.0 (-0.1, 2.1)	0.0 (-0.8, 0.7)	0.3 (-0.2, 0.8)	-0.3 (-0.9, 0.2)
**pgACC<->dACC_ISF2**	T0	1.6 ± 0.8	1.3 ± 0.6	1.1 ± 0.6	1.5 ± 1.2
Mean ± SD	T1	1.3 ± 0.9	1.1 ± 0.7	1.0 ± 0.7	1.4 ± 1.1
	T2	1.7 ± 1.2	1.2 ± 0.5	1.2 ± 0.7	1.6 ± 1.1
	T3	2.0 ± 1.3	1.2 ± 0.5	0.9 ± 0.6	1.4 ± 1.2
MD (95% CI)	T1-T0	-0.3 (-1.1, 0.5)	-0.1 (-0.6, 0.3)	-0.1 (-0.7, 0.5)	-0.1 (-0.5, 0.3)
	T2-T0	0.1 (-0.7, 0.9)	0.0 (-0.7, 0.6)	0.1 (-0.6, 0.8)	0.2 (-0.4, 0.7)
	T3-T0	0.4 (-0.3, 1.1)	-0.1 (-0.7, 0.5)	-0.3 (-0.8, 0.3)	-0.1 (-0.6, 0.4)
**pgACC<->dACC_ISF3**	T0	1.5 ± 1.1	1.3 ± 0.6	1.3 ± 0.4	1.1 ± 0.7
Mean ± SD	T1	1.7 ± 0.7	1.3 ± 0.6	1.2 ± 0.5	1.2 ± 0.6
	T2	1.5 ± 0.7	1.3 ± 1.0	1.3 ± 0.7	1.3 ± 0.8
	T3	1.6 ± 0.8	1.1 ± 0.9	1.1 ± 0.7	1.2 ± 0.7
MD (95% CI)	T1-T0	0.2 (-0.5, 0.9)	0.0 (-0.4, 0.4)	-0.1 (-0.5, 0.3)	0.0 (-0.3, 0.4)
	T2-T0	0.0 (-0.7, 0.7)	0.0 (-0.7, 0.6)	0.0 (-0.5, 0.5)	0.0 (-0.4, 0.3)
	T3-T0	0.1 (-0.6, 0.7)	-0.2 (-0.9, 0.5)	-0.2 (-0.8, 0.4)	0.0 (-0.3, 0.4)
**pgACC<->S1L_ISF1**	T0	1.9 ± 1.4	1.7 ± 1.0	1.4 ± 0.6	1.2 ± 0.6
Mean ± SD	T1	1.9 ± 1.0	1.8 ± 0.8	1.0 ± 0.3	1.4 ± 0.4
	T2	2.0 ± 1.0	1.7 ± 0.9	1.3 ± 0.7	1.2 ± 0.5
	T3	1.9 ± 0.7	1.4 ± 0.9	1.1 ± 0.4	1.2 ± 0.4
MD (95% CI)	T1-T0	0.0 (-0.9, 0.8)	0.1 (-0.7, 0.9)	-0.4 (-0.8, 0.0)	0.0 (-0.3, 0.3)
	T2-T0	0.1 (-1.0, 1.2)	0.0 (-0.8, 0.7)	-0.1 (-0.7, 0.5)	-0.1 (-0.6, 0.4)
	T3-T0	0.0 (-1.1, 1.0)	-0.3 (-1.3, 0.6)	-0.3 (-0.8, 0.1)	-0.2 (-0.7, 0.2)
**pgACC<->S1L_ISF2**	T0	1.8 ± 1.1	1.2 ± 0.6	1.3 ± 0.7	1.3 ± 0.8
Mean ± SD	T1	1.5 ± 0.9	1.3 ± 0.7	1.0 ± 0.5	1.2 ± 0.7
	T2	1.4 ± 0.7	1.0 ± 0.4	1.2 ± 0.5	1.5 ± 0.8
	T3	1.5 ± 0.7	1.1 ± 0.4	1.0 ± 0.6	1.3 ± 0.9
MD (95% CI)	T1-T0	-0.2 (-0.8, 0.3)	0.1 (-0.3, 0.5)	-0.4 (-1.0, 0.3)	0.0 (-0.3, 0.3)
	T2-T0	-0.4 (-1.1, 0.4)	-0.1 (-0.7, 0.4)	-0.2 (-0.8, 0.5)	0.2 (-0.4, 0.8)
	T3-T0	-0.3 (-1.0, 0.3)	-0.1 (-0.7, 0.5)	-0.3 (-0.9, 0.4)	0.0 (-0.6, 0.7)
**pgACC<->S1L_ISF3**	T0	1.1 ± 0.6	1.1 ± 0.7	0.8 ± 0.4	1.4 ± 0.6
Mean ± SD	T1	1.6 ± 0.7	1.2 ± 0.6	0.9 ± 0.6	1.1 ± 0.6
	T2	1.4 ± 0.6	1.2 ± 0.7	1.0 ± 0.6	1.1 ± 0.7
	T3	1.6 ± 0.7	1.0 ± 0.5	1.1 ± 0.6	1.2 ± 0.6
MD (95% CI)	T1-T0	0.5 (0.0, 1.0)	0.1 (-0.2, 0.5)	0.2 (-0.2, 0.5)	-0.3 (-0.8, 0.2)
	T2-T0	0.3 (-0.3, 0.9)	0.1 (-0.2, 0.4)	0.3 (-0.3, 0.8)	-0.3 (-0.8, 0.2)
	T3-T0	0.5 (-0.2, 1.2)	0.1 (-0.6, 0.6)	0.4 (-0.2, 0.9)	-0.2 (-0.7, 0.3)
**pgACC<->S1R_ISF1**	T0	1.7 ± 1.1	1.4 ± 0.6	1.4 ± 0.6	1.8 ± 1.0
Mean ± SD	T1	1.7 ± 0.8	1.4 ± 0.5	1.2 ± 0.6	1.5 ± 1.0
	T2	1.6 ± 0.7	1.3 ± 0.6	1.3 ± 0.5	1.3 ± 0.5
	T3	1.7 ± 0.9	1.3 ± 0.6	1.5 ± 1.1	1.5 ± 0.7
MD (95% CI)	T1-T0	0.0 (-0.7, 0.7)	0.0 (-0.6, 0.7)	-0.1 (-0.7, 0.4)	-0.2 (-0.7, 0.2)

Variable	Time point	Group 1 (pgACC)	Group 2 (dACC+SSC)	Group 3 (Ratio)	Group 4 (Placebo)
	T2-T0	0.0 (-0.9, 0.8)	-0.1 (-0.8, 0.6)	-0.1 (-0.5, 0.4)	-0.5 (-1.2, 0.2)
	T3-T0	0.0 (-1.0, 1.0)	-0.1 (-0.5, 0.4)	0.1 (-0.8, 1.1)	-0.2 (-1.0, 0.6)
**pgACC<->S1R_ISF2** Mean ± SD	T0	1.2 ± 0.7	1.2 ± 0.7	1.1 ± 0.5	1.0 ± 0.5
	T1	1.5 ± 0.7	1.3 ± 0.6	1.1 ± 0.6	0.7 ± 0.4
	T2	1.2 ± 0.8	1.1 ± 0.7	1.0 ± 0.5	0.8 ± 0.3
	T3	1.2 ± 0.6	1.1 ± 0.7	1.4 ± 0.9	0.9 ± 0.5
MD (95% CI)	T1-T0	0.3 (0.0, 0.6)	0.1 (-0.2, 0.4)	0.1 (-0.4, 0.5)	-0.3 (-0.6, 0.0)
	T2-T0	0.1 (-0.6, 0.7)	-0.1 (-0.5, 0.3)	-0.1 (-0.7, 0.5)	-0.2 (-0.6, 0.2)
	T3-T0	0.1 (-0.6, 0.7)	-0.1 (-0.5, 0.4)	0.3 (-0.3, 0.9)	-0.1 (-0.6, 0.5)
**pgACC<->S1R_ISF3** Mean ± SD	T0	1.6 ± 0.9	1.5 ± 0.5	0.8 ± 0.2	0.9 ± 0.7
	T1	1.7 ± 1.1	1.1 ± 0.7	0.9 ± 0.3	1.2 ± 0.7
	T2	1.7 ± 1.1	1.1 ± 0.6	1.3 ± 0.7	0.9 ± 0.4
	T3	2.1 ± 1.1	1.1 ± 0.7	1.2 ± 0.5	1.1 ± 0.3
MD (95% CI)	T1-T0	0.1 (-0.3, 0.5)	-0.4 (-0.8, 0.1)	0.1 (-0.1, 0.3)	0.0 (-0.5, 0.6)
	T2-T0	0.1 (-0.6, 0.9)	-0.4 (-0.7, 0.0)	0.4 (0.0, 0.9)	-0.2 (-0.8, 0.4)
	T3-T0	0.4 (-0.3, 1.1)	-0.4 (-0.7, -0.1)	0.4 (0.2, 0.7)	0.0 (-0.4, 0.4)
**dACC<->S1L_ISF1** Mean ± SD	T0	1.8 ± 0.8	1.5 ± 0.7	1.8 ± 1.0	1.5 ± 0.8
	T1	1.6 ± 0.8	1.7 ± 0.6	1.4 ± 0.8	1.3 ± 0.7
	T2	1.7 ± 0.5	2.0 ± 1.3	1.4 ± 0.5	1.3 ± 0.5
	T3	1.9 ± 0.7	1.8 ± 1.3	1.4 ± 0.5	1.2 ± 0.6
MD (95% CI)	T1-T0	-0.2 (-0.9, 0.6)	0.2 (-0.2, 0.6)	-0.4 (-1.0, 0.2)	-0.1 (-0.9, 0.7)
	T2-T0	-0.1 (-0.6, 0.5)	0.5 (-0.6, 1.5)	-0.4 (-1.1, 0.3)	-0.2 (-0.8, 0.4)
	T3-T0	0.1 (-0.5, 0.7)	0.3 (-0.6, 1.2)	-0.4 (-1.1, 1.4)	-0.2 (-0.8, 0.3)
**dACC<->S1L_ISF2** Mean ± SD	T0	1.9 ± 1.1	1.2 ± 0.5	1.2 ± 0.5	1.2 ± 0.7
	T1	1.9 ± 0.8	1.2 ± 0.6	1.2 ± 0.6	1.1 ± 0.5
	T2	1.7 ± 0.7	0.9 ± 0.3	1.0 ± 0.5	1.4 ± 0.6
	T3	1.6 ± 0.8	1.2 ± 0.6	1.2 ± 0.7	1.3 ± 0.5
MD (95% CI)	T1-T0	-0.1 (-0.8, 0.7)	0.0 (-0.4, 0.3)	0.0 (-0.1, 0.1)	-0.1 (-0.4, 0.2)
	T2-T0	-0.2 (-0.9, 0.5)	-0.3 (-0.7, 0.1)	-0.1 (-0.5, 0.3)	0.0 (-0.4, 0.4)
	T3-T0	-0.3 (-1.1, 0.4)	0.0 (-0.6, 0.5)	0.0 (-0.6, 0.7)	-0.1 (-0.6, 0.4)
**dACC<->S1L_ISF3** Mean ± SD	T0	1.4 ± 0.8	1.1 ± 0.4	1.3 ± 0.4	1.4 ± 1.3
	T1	1.7 ± 0.7	1.0 ± 0.4	1.0 ± 0.5	1.5 ± 1.4
	T2	1.6 ± 0.7	1.2 ± 0.8	1.1 ± 0.6	1.4 ± 1.3
	T3	1.7 ± 0.8	0.8 ± 0.5	1.1 ± 0.7	1.8 ± 1.2
MD (95% CI)	T1-T0	0.3 (-0.2, 0.7)	-0.1 (-0.5, 0.3)	-0.3 (-0.6, 0.1)	0.0 (-0.5, 0.6)
	T2-T0	0.2 (-0.5, 1.0)	0.1 (-0.6, 0.7)	-0.2 (-0.6, 0.3)	0.0 (-0.5, 0.5)
	T3-T0	0.3 (-0.3, 0.9)	-0.3 (-0.8, 0.3)	-0.1 (-0.6, 0.3)	0.4 (-0.1, 0.9)
**dACC<->S1R_ISF1** Mean ± SD	T0	1.8 ± 0.9	1.6 ± 0.7	1.5 ± 0.7	1.5 ± 0.7
	T1	2.3 ± 1.8	1.7 ± 1.1	1.5 ± 0.6	1.4 ± 0.7
	T2	2.1 ± 1.0	1.5 ± 1.0	1.3 ± 0.5	1.1 ± 0.6
	T3	2.0 ± 1.0	1.7 ± 0.7	1.5 ± 0.8	1.3 ± 0.7
MD (95% CI)	T1-T0	0.5 (-0.6, 1.7)	0.1 (-0.9, 1.1)	0.0 (-0.5, 0.5)	-0.1 (-0.4, 0.2)
	T2-T0	0.3 (-0.5, 1.0)	-0.1 (-0.8, 0.6)	-0.2 (-0.8, 0.4)	-0.5 (-1.1, 0.2)
	T3-T0	0.2 (-0.8, 1.2)	0.0 (-0.6, 0.7)	0.0 (-0.7, 0.6)	-0.2 (-0.9, 0.6)
**dACC<->S1R_ISF2** Mean ± SD	T0	1.3 ± 0.5	1.0 ± 0.4	1.3 ± 0.5	1.2 ± 0.5
	T1	1.3 ± 0.8	1.3 ± 0.5	1.3 ± 0.8	1.1 ± 0.3
	T2	1.6 ± 1.0	1.1 ± 0.6	1.4 ± 0.9	1.2 ± 0.8
	T3	1.4 ± 0.4	1.4 ± 1.0	1.3 ± 0.6	1.3 ± 0.7
MD (95% CI)	T1-T0	0.0 (-0.6, 0.6)	0.2 (-0.2, 0.7)	0.0 (-0.5, 0.6)	-0.1 (-0.5, 0.4)
	T2-T0	0.3 (-0.3, 0.9)	0.1 (-0.3, 0.4)	0.1 (-0.7, 0.9)	0.1 (-0.6, 0.7)

Variable	Time point	Group 1 (pgACC)	Group 2 (dACC+SSC)	Group 3 (Ratio)	Group 4 (Placebo)
	T3-T0	0.2 (-0.3, 0.6)	0.3 (-0.3, 1.0)	0.0 (-0.7, 0.7)	0.2 (-0.6, 0.9)
**dACC<->S1R_ISF3**	T0	1.4 ± 0.8	1.1 ± 0.7	1.3 ± 0.6	1.5 ± 0.9
Mean ± SD	T1	1.2 ± 0.7	1.1 ± 0.6	1.2 ± 0.5	1.4 ± 1.0
	T2	1.9 ± 1.7	0.8 ± 0.4	1.1 ± 0.7	1.0 ± 0.5
	T3	1.7 ± 1.5	0.8 ± 0.4	1.0 ± 0.6	1.2 ± 0.7
MD (95% CI)	T1-T0	-0.2 (-0.5, 0.2)	0.0 (-0.5, 0.4)	-0.1 (-0.4, 0.3)	-0.2 (-0.7, 0.4)
	T2-T0	0.5 (-0.4, 1.4)	-0.3 (-1.0, 0.3)	-0.1 (-0.6, 0.4)	-0.6 (-1.3, 0.2)
	T3-T0	0.3 (-0.6, 1.1)	-0.3 (-0.7, 0.0)	-0.3 (-0.7, 0.2)	-0.3 (-0.6, 0.1)
**S1L<->S1R_ISF1**	T0	1.3 ± 0.5	1.2 ± 0.6	1.7 ± 0.7	1.7 ± 0.7
Mean ± SD	T1	1.9 ± 0.9	1.2 ± 0.5	1.6 ± 0.5	1.3 ± 0.5
	T2	2.2 ± 1.0	1.7 ± 0.8	1.8 ± 0.8	1.5 ± 0.5
	T3	1.8 ± 1.1	1.5 ± 0.8	1.8 ± 1.1	1.3 ± 0.5
MD (95% CI)	T1-T0	0.6 (0.0, 1.2)	0.0 (-0.5, 0.5)	-0.2 (-0.7, 0.4)	-0.4 (-1.1, 0.3)
	T2-T0	0.9 (0.2, 1.5)	0.5 (-0.1, 1.1)	0.0 (-0.8, 0.8)	-0.2 (-0.9, 0.5)
	T3-T0	0.5 (-0.2, 1.2)	0.3 (-0.4, 1.0)	0.1 (-1.0, 1.1)	-0.4 (-1.0, 0.2)
**S1L<->S1R_ISF2**	T0	1.7 ± 0.8	1.3 ± 0.5	1.2 ± 0.7	1.1 ± 0.5
Mean ± SD	T1	1.6 ± 0.9	1.3 ± 0.4	1.1 ± 0.7	0.9 ± 0.4
	T2	1.9 ± 1.2	1.2 ± 0.4	1.1 ± 0.5	1.0 ± 0.5
	T3	1.3 ± 0.9	1.3 ± 0.5	1.2 ± 0.8	1.3 ± 0.5
MD (95% CI)	T1-T0	-0.1 (-0.7, 0.5)	0.0 (-0.4, 0.4)	-0.1 (-0.4, 0.2)	-0.1 (-0.4, 0.2)
	T2-T0	0.1 (-0.7, 1.0)	-0.1 (-0.6, 0.4)	-0.3 (-0.7, 0.1)	0.0 (-0.4, 0.4)
	T3-T0	-0.4 (-1.1, 0.2)	0.0 (-0.5, 0.5)	-0.1 (-0.7, 0.6)	0.3 (-0.2, 0.8)
**S1L<->S1R_ISF3**	T0	1.4 ± 0.8	1.5 ± 0.6	1.2 ± 0.6	1.5 ± 1.0
Mean ± SD	T1	1.5 ± 0.8	1.4 ± 0.6	1.2 ± 0.5	1.2 ± 1.0
	T2	1.6 ± 0.9	1.0 ± 0.8	1.0 ± 0.5	1.1 ± 0.7
	T3	1.7 ± 1.0	1.0 ± 0.7	1.1 ± 0.5	1.2 ± 0.6
MD (95% CI)	T1-T0	0.1 (-0.7, 0.9)	-0.1 (-0.4, 0.1)	0.0 (-0.4, 0.5)	-0.3 (-1.2, 0.6)
	T2-T0	0.1 (-0.6, 0.9)	-0.5 (-0.9, -0.1)	-0.2 (-0.7, 0.3)	-0.4 (-1.2, 0.4)
	T3-T0	0.3 (-0.5, 1.1)	-0.5 (-0.9, -0.1)	-0.1 (-0.8, 0.5)	-0.3 (-1.0, 0.3)

CI: Confidence Interval, dACC: dorsal anterior cingulate cortex, ISF1: Infraslow frequency- low band, ISF2: Infraslow frequency- mid band, ISF3: Infraslow frequency- high band, MD: Mean Difference, pgACC: pregenual anterior cingulate cortex, SD: Standard Deviation, S1L: Primary Somatosensory cortex left, S1R: Primary Somatosensory cortex right, T0: Baseline, T1: Immediately post-treatment, T2: One week follow up, T3: One month follow up.

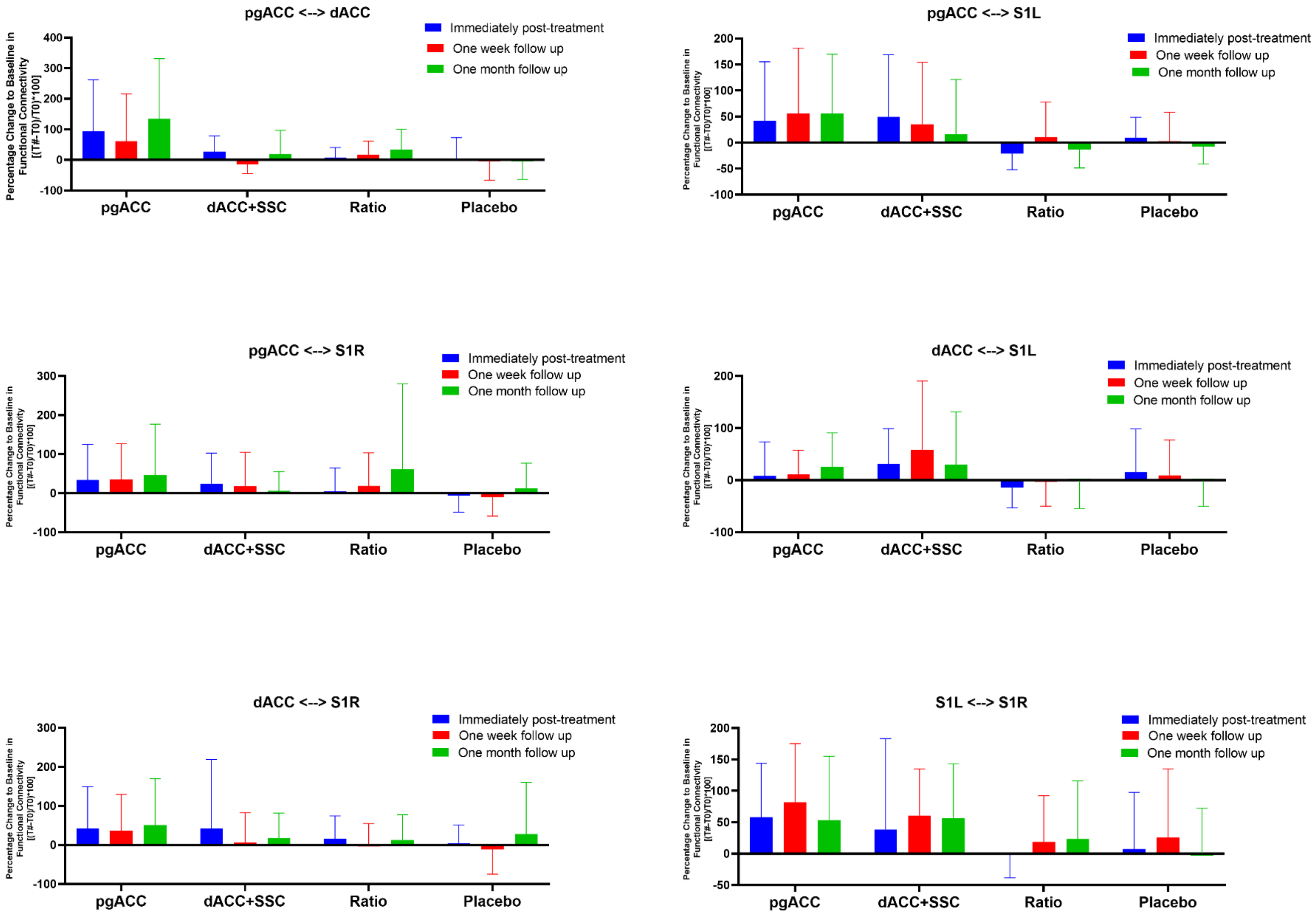

Supplementary Figure S4. Percentage changes to baseline in functional connectivity between the targeted regions in ISF1 band.

The bar represents the Mean, and the error bars represent the standard deviations. pgACC: pregenual anterior cingulate cortex, dACC: dorsal anterior cingulate cortex, S1L: left primary somatosensory cortex, S1R: right primary somatosensory cortex, <--->: Functional connectivity.

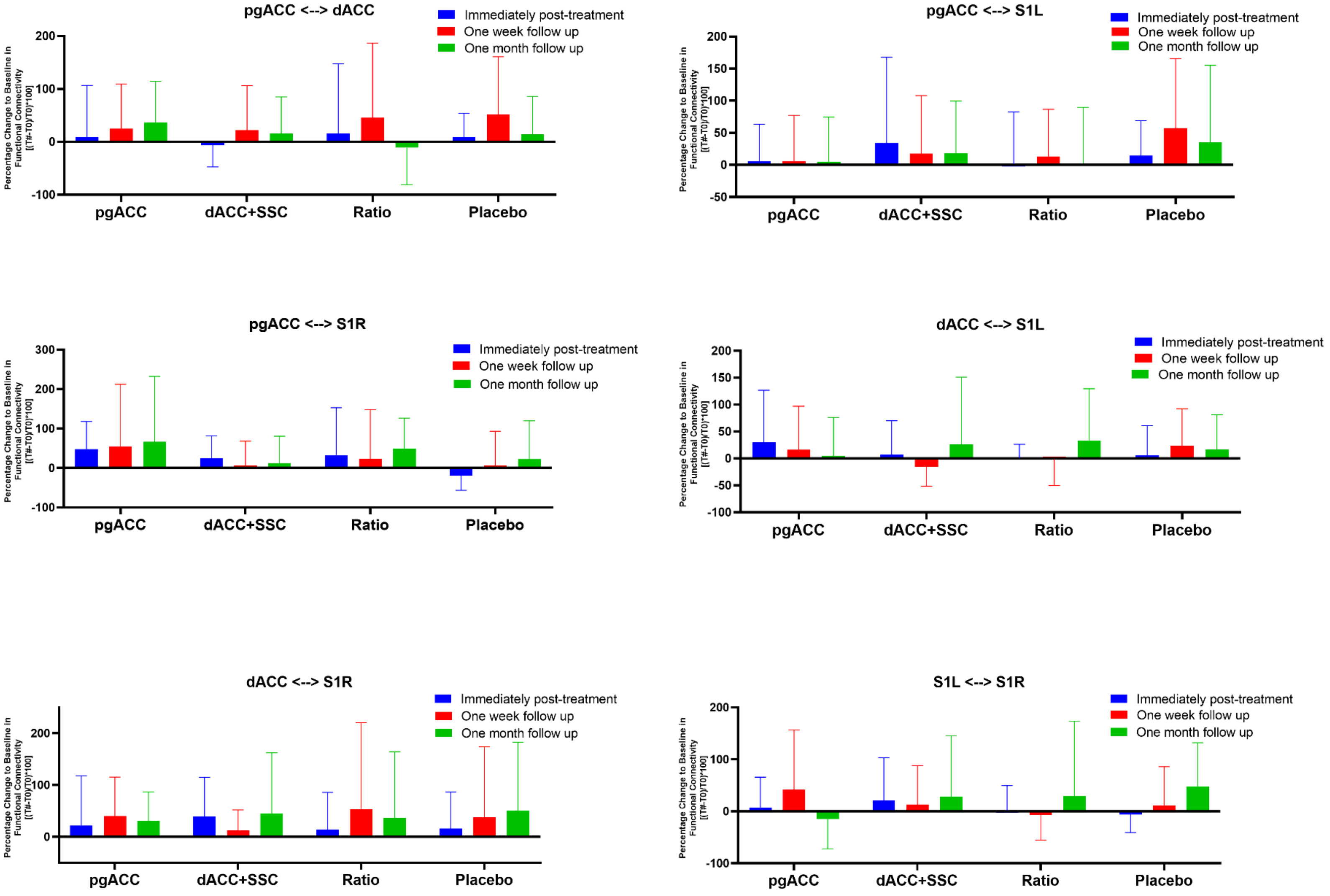

Supplementary Figure S5. Percentage changes to baseline in functional connectivity between the targeted regions in ISF2 band.

The bar represents the Mean, and the error bars represent the standard deviations. pgACC: pregenual anterior cingulate cortex, dACC: dorsal anterior cingulate cortex, S1L: left primary somatosensory cortex, S1R: right primary somatosensory cortex, <--->: Functional connectivity.

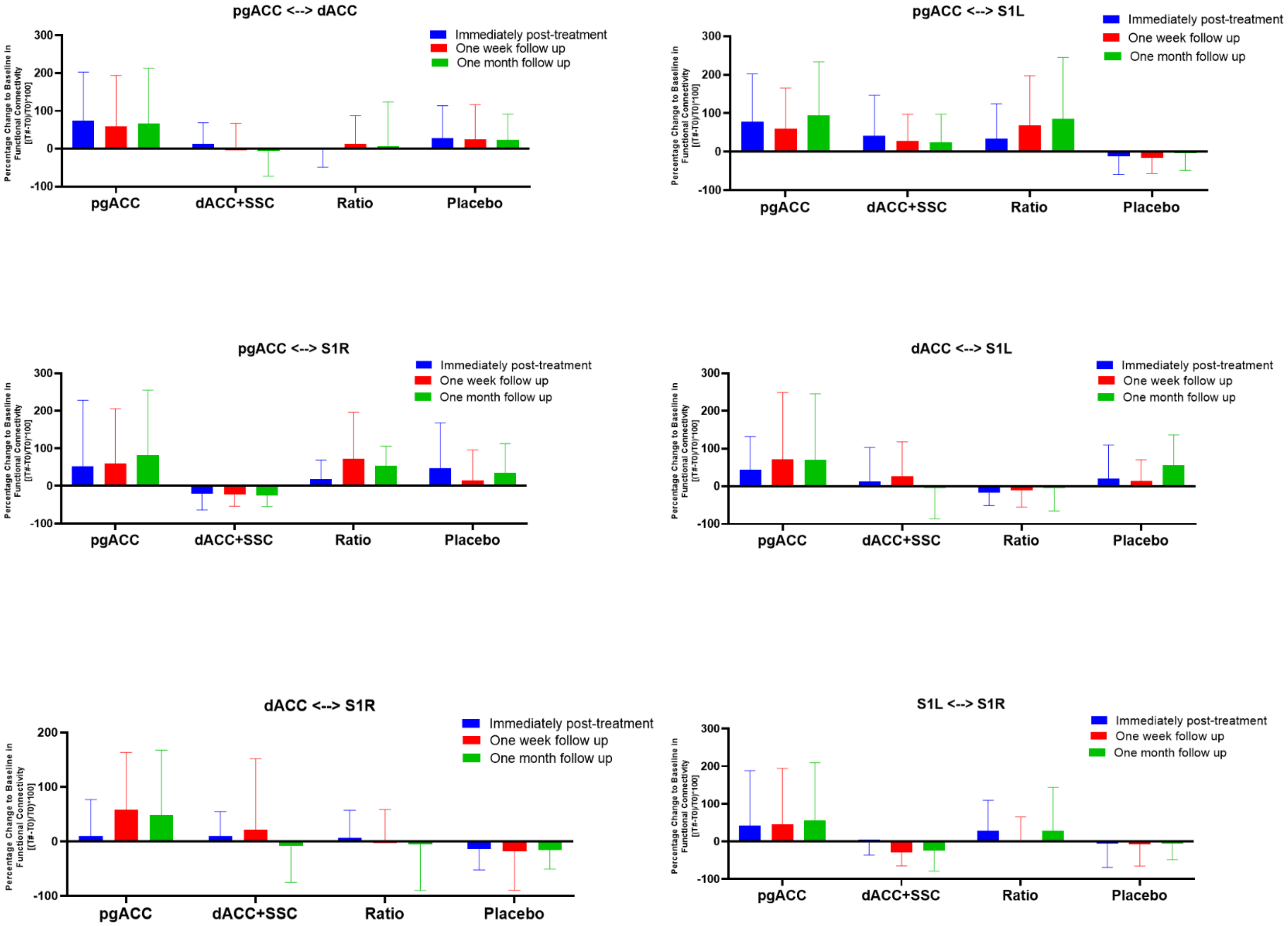

Supplementary Figure S6. Percentage changes to baseline in functional connectivity between the targeted regions in ISF3 band.

The bar represents the Mean, and the error bars represent the standard deviations. pgACC: pregenual anterior cingulate cortex, dACC: dorsal anterior cingulate cortex, S1L: left primary somatosensory cortex, S1R: right primary somatosensory cortex, <--->: Functional connectivity.
